# Breast milk and in utero transmission of HIV-1 select for envelope variants with unique molecular signatures

**DOI:** 10.1186/s12977-017-0331-z

**Published:** 2017-01-26

**Authors:** Kyle J. Nakamura, Laura Heath, Edwin R. Sobrera, Thomas A. Wilkinson, Katherine Semrau, Chipepo Kankasa, Nicole H. Tobin, Nicholas E. Webb, Benhur Lee, Donald M. Thea, Louise Kuhn, James I. Mullins, Grace M. Aldrovandi

**Affiliations:** 1 Division of Infectious Diseases, Children’s Hospital Los Angeles, Los Angeles, CA USA; 2 Systems Biology and Disease Program, USC Keck School of Medicine, Los Angeles, CA USA; 3 Department of Microbiology, University of Washington, Seattle, WA USA; 4 Division of Global Health Equity, Brigham and Women’s Hospital, Harvard Medical School, and Ariadne Labs, Boston, MA USA; 5 University Teaching Hospital, University of Zambia, Lusaka, Zambia; 6 Division of Pediatric Infectious Diseases, Department of Pediatrics, David Geffen School of Medicine at the University of California at Los Angeles, Los Angeles, CA USA; 7 Department of Microbiology, Icahn School of Medicine at Mount Sinai, New York, NY USA; 8 Department of Epidemiology, Columbia University, New York, NY USA

**Keywords:** HIV-1, Mother-to-child transmission, Envelope, CD4, Glycosylation, Broadly neutralizing antibodies

## Abstract

**Background:**

Mother-to-child transmission of human immunodeficiency virus-type 1 (HIV-1) poses a serious health threat in developing countries, and adequate interventions are as yet unrealized. HIV-1 infection is frequently initiated by a single founder viral variant, but the factors that influence particular variant selection are poorly understood.

**Results:**

Our analysis of 647 full-length HIV-1 subtype C and G viral envelope sequences from 22 mother–infant pairs reveals unique genotypic and phenotypic signatures that depend upon transmission route. Relative to maternal strains, intrauterine HIV transmission selects infant variants that have shorter, less-glycosylated V1 loops that are more resistant to soluble CD4 (sCD4) neutralization. Transmission through breastfeeding selects for variants with fewer potential glycosylation sites in gp41, are more sensitive to the broadly neutralizing antibodies PG9 and PG16, and that bind sCD4 with reduced cooperativity. Furthermore, experiments with Affinofile cells indicate that infant viruses, regardless of transmission route, require increased levels of surface CD4 receptor for productive infection.

**Conclusions:**

These data provide the first evidence for transmission route-specific selection of HIV-1 variants, potentially informing therapeutic strategies and vaccine designs that can be tailored to specific modes of vertical HIV transmission.

**Electronic supplementary material:**

The online version of this article (doi:10.1186/s12977-017-0331-z) contains supplementary material, which is available to authorized users.

## Background

Despite focused efforts upon preventative measures, pediatric human immunodeficiency virus-type 1 (HIV-1) infections through mother-to-child transmission (MTCT) continue to challenge clinicians and strain healthcare systems, with 1.5 million HIV-positive women giving birth and 240,000 children acquiring the virus in 2013 [1]. HIV-1 MTCT can occur through three distinct routes: in utero (transplacental passage), intrapartum, and postpartum through breastfeeding. Studies of HIV-infected infants indicate that disease progression can vary dramatically among these transmission modes, with infants infected in utero showing the shortest times of survival [2, 3]. An understanding of the genotypic and phenotypic factors that uniquely distinguish these routes of HIV transmission is crucial for developing targeted preventative strategies including vaccines.

A large majority of HIV-1 infections that occur through sexual or vertical transmission are established by a single “founder” variant, with primary infection being characterized by a virus population with significantly less sequence diversity compared to donor viruses [4–17]. The founding variant(s) typically utilize the chemokine receptor CCR5 for entry, and may represent a minority population from the donor [4, 8–10, 14, 18–22]. Studies of heterosexual transmission (subtypes A, C and D) [5, 18, 23, 24] and of MTCT (subtype CRF01_AE) [12] have identified certain “molecular signatures” in founder variants, such as shorter envelope variable loops, loss of potential *N*-glycosylation sites (PNGs), or selection of particular PNGs in transmitted strains, but no such signatures have been observed following transmission of subtype B variants [23, 25, 26]. Although previous MTCT studies had limitations of small sample size, a limited focus to specific regions within the envelope, and/or the inability to discriminate between different routes of MTCT [4, 6, 7, 9, 12, 13, 27–33], all of these studies reported detection of a single or few founder variants. Some (but not all) MTCT studies have reported genetic differences between viral strains transmitted in utero and intrapartum [6, 12, 34]. Investigations of the role of maternal neutralizing antibodies in shaping the founder virus population during MTCT have yielded conflicting results: while some data show that selected founder viruses represent maternal antibody escape variants [30, 34], other studies find no such relationship [32].

As the major viral protein assembly on the virion surface, the HIV envelope protein spike holds the dual distinction of being both the ‘key’ that the virus uses to unlock susceptible target cells in the exposed infant as well as being the primary target of neutralizing antibodies. The HIV-1 envelope spike assembly is composed of a trimer of glycosylated gp120/gp41 heterodimers, and is responsible for mediating entry into host cells via interactions with the CD4 receptor and a co-receptor (typically CCR5). The gp120 subunit lies entirely on the extracellular surface, while the standard envelope topology model describes gp41 as having an extracellular domain, a single membrane-spanning domain, and a cytoplasmic domain (CD) [35]. Analysis of gp120 sequences from diverse viral strains identifies five regions of high sequence variability (variable domains V1–V5) interspersed within five relatively constant domains (C1–C5) [36, 37] (Additional file 1: Figure S1). The tremendous sequence diversity within the gp120 variable regions enables escape from neutralizing antibodies [38]. In addition to CD4 and CCR5, the gp120 subunit can engage other cell-surface proteins that may facilitate mucosal transmission, such as dendritic cell-specific intercellular adhesion molecule-3-grabbing non-integrin (DC-SIGN) [39] and the integrin α_4_β_7_ [40, 41]. Envelope recognition of the CD4 receptor, co-receptor, and other key cell surface proteins, all while simultaneously escaping antibody surveillance, suggests functional sequence constraints that govern the selection of the founding variant(s) from the swarm of donor isolates during transmission.

MTCT differs significantly from other HIV transmission modes: first, MTCT occurs in the presence of maternal antibodies to HIV, with infant levels of maternal antibodies being greater than or equivalent to those in the mother at birth, and then decreasing over the first few months of life [42]. Second, the infant immune system differs in the quantity, distribution, and activation state of CD4^+^ T cells compared to adults [43]. Third, infants share half their genetic identity with their mothers, leading to greater immunologic overlap between donor and recipient than occurs during sexual or parenteral transmission. Moreover, MTCT in utero, intrapartum and through breastfeeding each involves unique physical exposures, biological barriers, and developmental states of the infant that could influence transmission dynamics and founding strain selection. Given such distinctive transmission features, as well as the critical role of HIV envelope protein in establishing infection and escaping the host immune system, we performed a large, systematic analysis of the genetic and phenotypic properties of HIV envelope variants transmitted from mother to child by different routes, with the aim of identifying viral characteristics that might confer selective advantages for mode-specific transmission.

## Results

### Transmission timing and phylogenetic linkage of HIV strains within mother-to-child transmission pairs

**Fig. 1:**
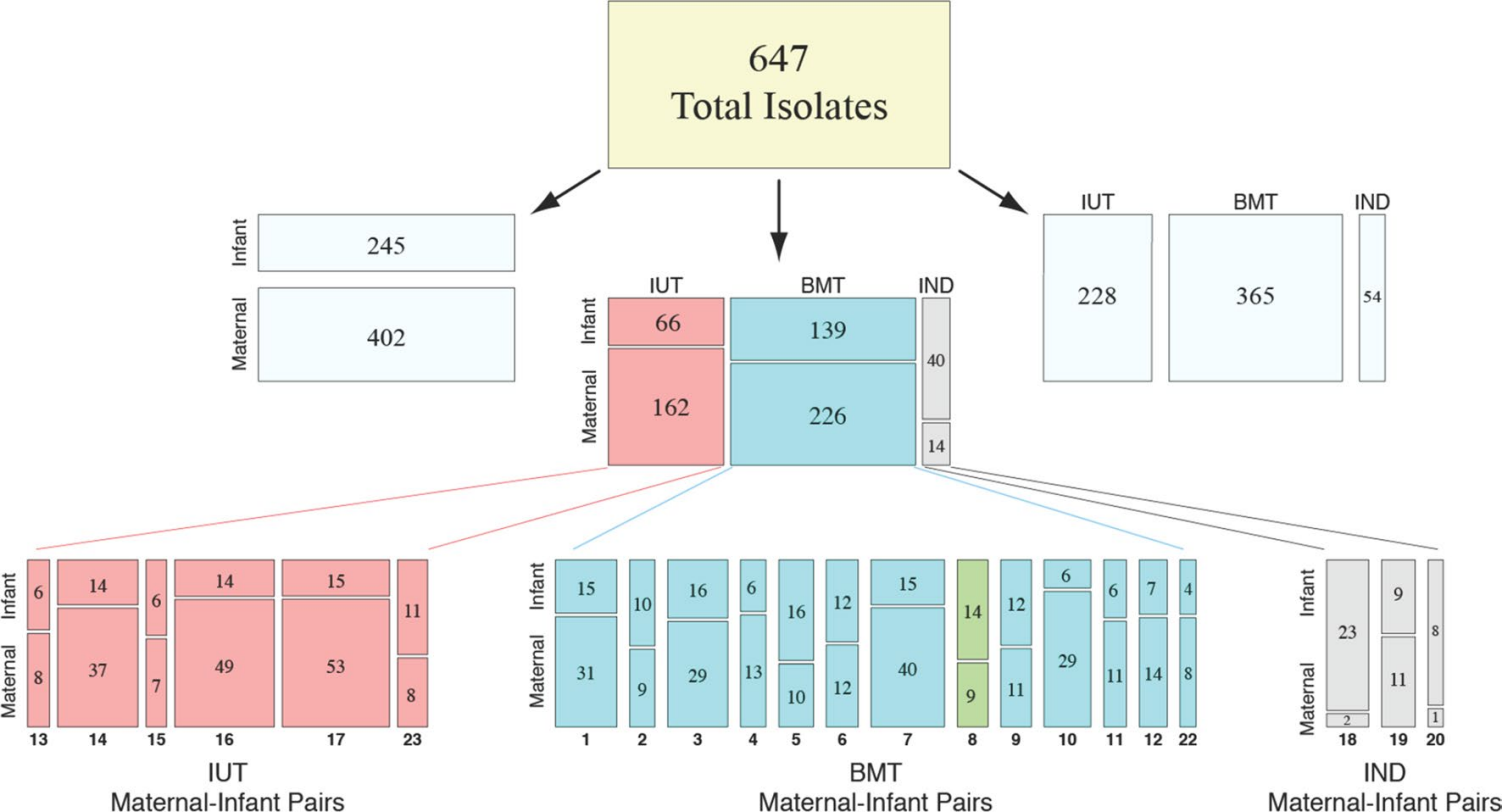
Mosaic plots showing groupings by subject (maternal or infant), transmission mode, and transmission pair ID number for the 647 infant- and maternal-derived envelope clones obtained in this study. The number of envelope variants represented within each group is indicated inside each tile. IUT, in utero transmission; BMT, breast milk transmission; IND, indeterminate transmission mode. Twenty-one transmission pairs were infected with HIV-1 subtype C, while one pair (Pair 8, shown in *green*) was infected with subtype G

**Fig. 2:**
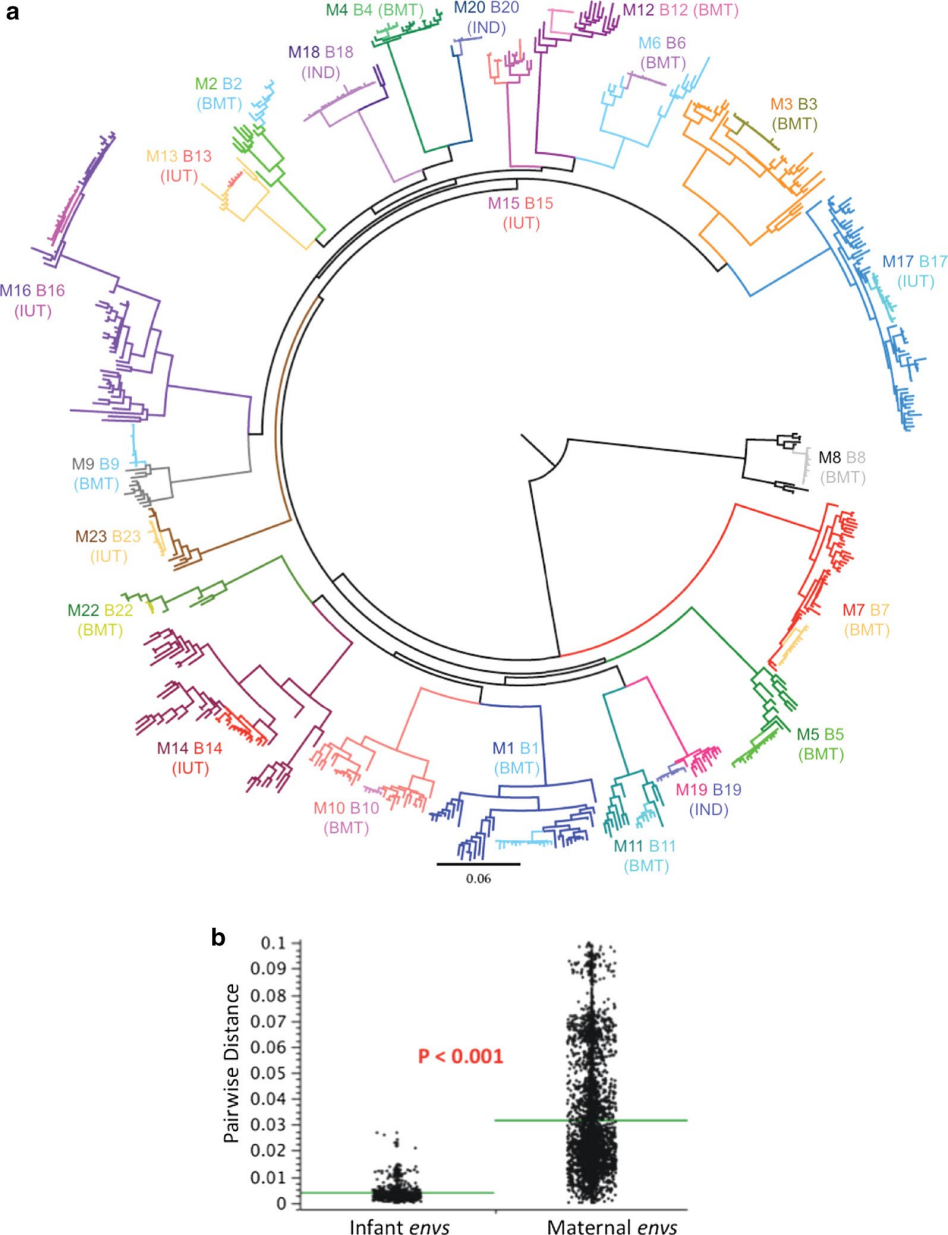
Genetic analysis of HIV envelope sequences among MTCT pairs. **a** Phylogenetic tree of all 22 mother–infant transmission pairs included in study. All maternal envelopes form distinct phylogenetic clusters, with infant envelopes forming a subcluster within the larger maternal cluster. Infant envelopes are colored differently from the maternal envelopes (often in a *lighter shade*). **b** Comparison of infant versus maternal envelope diversity was performed using the approach described by Gilbert et al. [104] for comparing genetic distances. Infant envelopes (shown on the *left*) were significantly less diverse than maternal envelopes (p < 0.001)

We examined 22 MTCT pairs participating in the Zambia Exclusive Breastfeeding Study (ZEBS) [44]. Six mothers from this cohort transmitted in utero (IUT), as their infants were HIV-positive at birth by PCR tests, while 13 mothers transmitted virus through breastfeeding (BMT), since their infants were HIV-negative at birth and at 1 month of age, but HIV-positive after 42 days. Three mothers were classified as “indeterminate” transmitters (IND), as their infants were HIV-negative at birth but HIV-positive after 1 month: as such, the precise timing of when these infants acquired virus (either postpartum or during labor and delivery) is unclear. To confirm transmission linkage, we cloned, sequenced and compared 647 envelope genes following polymerase chain reaction (PCR) amplification. In all, we obtained 245 envelope sequences from infants from the first available HIV-1 PCR-positive infant blood sample, and 402 sequences from mothers (Fig. 1). Envelope sequences of maternal origin were typically taken from maternal blood and breast milk samples collected either at study entry (for IUT and IND pairs) or from maternal samples collected within ~35 days prior to the infant’s first positive PCR test (for BMT pairs) (Table 1). In all, we obtained 228 envelope sequences from the IUT group, 365 sequences from the BMT group, and 54 sequences from the IND group (Fig. 1). From within the IUT group, 162 envelope sequences were amplified from blood samples and 66 from milk, while from within the BMT group 309 and 56 envelope sequences were obtained from blood and milk samples, respectively (Table 2). When gp160 sequences from the entire cohort were placed into a phylogenetic tree, each transmission pair formed a monophyletic cluster, with infant envelope sequences forming a sub-cluster within each larger maternal cluster (Fig. 2a). From this analysis, we determined that 21 transmission pairs were infected with subtype C virus, while one pair (Pair 8 from the BMT group) was infected with HIV-1 subtype G (Fig. 1).

**Table 1:** Clinical data for the 22 maternal–infant pairs in the cohort

Route of transmission	Maternal–infant pair ID	Maternal HIV RNA (copies/mL of plasma)	Maternal CD4^+^ count (cells/nL)	Time between infant and maternal sample collection (days)
BMT	1	300,000	76	158
BMT	2	200,656	154	240
BMT	3	206,763	94	143
BMT	4	50,291	332	205
BMT	5	298,310	110	35
BMT	6	143,217	276	35
BMT	7	270,691	317	31
BMT	8	117,007	103	28
BMT	9	104,209	198	32
BMT	10	509,607	300	37
BMT	11	83,060	137	35
BMT	12	45,907	52	35
BMT	22	43,301	88	38
	*Median*	143,217	137	35
IUT	13	39,801	138	91
IUT	14	375,319	94	99
IUT	15	18,220	246	350
IUT	16	211,792	118	191
IUT	17	47,602	291	36
IUT	23	537,736	318	183
	*Median*	129,697	192	141
IND	18	750,001	91	227
IND	19	358,315	219	132
IND	20	6855	299	140
	*Median*	358,315	219	140

**Table 2:** Number of envelope sequences obtained from either blood or milk samples, grouped by transmission mode

Route of transmission	Source of *env* sequences	
	Blood	Milk
IUT	162	66
BMT	309	56
IND	53	1

*IUT* in utero transmission, *BMT* transmission through breastfeeding, *IND* indeterminate (timing of HIV transmission unclear)

### Founder populations and reduction in viral diversity upon mother-to-child transmission

**Fig. 3:**
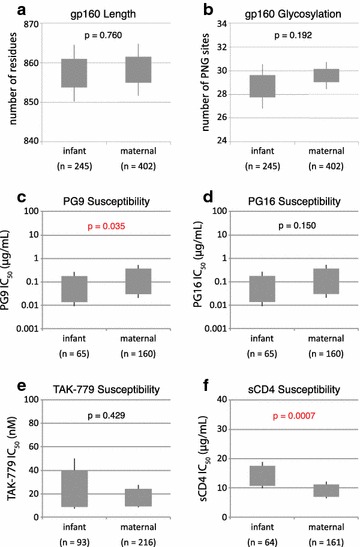
Aggregate analysis of HIV envelope proteins from the 22 transmission pairs included in this study. GEE-derived p values indicate whether genotypic and phenotypic parameters are significantly different between infant and maternal groups, with p values ≤0.05 shown in *red*. The *gray box* indicates the 95% confidence interval and the *whiskers* indicate the 99% confidence interval. **a** Comparison of gp160 length (in number of amino acids) for all maternal and infant envelopes. **b** Comparison of gp160 glycosylation level (in number of PNG motifs) for maternal and infant envelopes. **c**–**f** Comparison of the mean 50% inhibitory concentration (IC_50_) of **c** PG9, **d** PG16, **e** TAK-779, and **f** sCD4 against envelopes from maternal and infant isolates. The number of envelope sequences tested for each group (n) is also shown

Envelope amino acid sequences obtained from infant samples were significantly less diverse than maternal sequences (Fig. 2b), but did not differ significantly in gp160 length (mean of 858.2 residues for all infant strains in the cohort, and 857.4 residues for the maternal strains; p = 0.760) or in number of PNGs (mean of 29.6 and 28.7 PNGs for infant and maternal strains, respectively; p = 0.192), as determined by comparison of means derived from generalized estimating equations (GEE) modeling (Fig. 3a, b). The number of founder variants establishing infection was judged by analyzing phylogenies, viral diversity, insertion and deletion patterns (indels) and the number and linkage of phylogenetically informative mutations in each infant’s virus population (see Additional file 10: Table S1; Additional file 2: Figures S2, Additional file 3: Figure S3). At least 12/22 infections (9/13 BMT, 2/6 IUT, and 1/3 IND) were consistent with a single founder variant. The incidence of putative multi-founder infections was lower in the BMT group than in the IUT group (31% of breast milk transmissions vs. 67% of in utero transmissions), but this difference was not statistically significant (p = 0.319, Fisher’s exact test).

### Distinct envelope protein characteristics of maternal and infant virus variants

All infant variants used CCR5 for entry, as determined from infection assays using pseudotyped HIV virions bearing the infant envelope sequences and TZM-bl cells in the presence of high TAK-779 concentrations, and/or from infection of GHOST cells co-expressing CD4 and either CCR5 or CXCR4 (data not shown).

To determine if a critical set of epitopes within HIV-1 envelopes are being selected during MTCT in this cohort, we tested a broad array of envelope-specific inhibitors in infection assays against pseudotyped HIV-1 virions bearing viral envelopes of either infant or maternal origin. These inhibitors included broadly neutralizing monoclonal antibodies (bnAbs) such as the V1/V2 loop-specific PG9 and PG16 [45–47]; 2F5 and 4E10, which bind to the membrane-proximal external region (MPER) of gp41 [48–51]; 2G12, which recognizes carbohydrate moieties on the outer domain of gp120 [48, 52, 53]; and b12, which targets a CD4 binding epitope on gp120 [54–58]. We also tested the inhibitory activities of soluble sCD4 (a 26 kDa protein) [59], the small molecule CCR5 inhibitor TAK-779 [60, 61], and the 36-amino acid fusion inhibitor enfuvirtide (T-20) [62], which we used to probe HIV-1 envelope interactions with the CD4 receptor, the CCR5 co-receptor, and the envelope capacity to trigger membrane fusion, respectively. These experiments allowed (a) probing of neutralization sensitivity using standardized reagents, (b) examination of the accessibility of specific neutralizing epitopes (e.g., the gp41 MPER), and (c) assessment of the potential usefulness of available bnAbs in the setting of MTCT (either as a prophylactic intervention or a vaccine template).

In all, we performed inhibition assays with 322 different envelopes from this cohort: 223 obtained from maternal samples and 99 from infant samples. Susceptibility to the bnAbs 4E10, 2F5, b12, 2G12, and T-20 was similar between infant and maternal variants (data not shown). Infant variants also showed similar susceptibility as the maternal variants to PG16 (p = 0.150) and TAK-779 (p = 0.429), but were more sensitive to PG9 (mean IC_50_ of 0.17 µg/mL for infant strains and 0.38 µg/mL for maternal strains; p = 0.035) and more resistant than the maternal variants to sCD4 (mean IC_50_ of 13.7 µg/mL for infant strains and 8.8 µg/mL for maternal strains; p = 0.0007) (Fig. 3c–f). When the data were stratified by route of transmission, a unique transmission signature was identified for each route, as described below. To better examine genotypic and phenotypic differences in strains having precisely defined modes of transmission, we focused our analysis on strains solely from the IUT and BMT groups, and did not explore further the envelope variants from the IND group.

### ‘Transmission signatures’ of in utero and breast milk infection

#### Genotypic characteristics

**Fig. 4:**
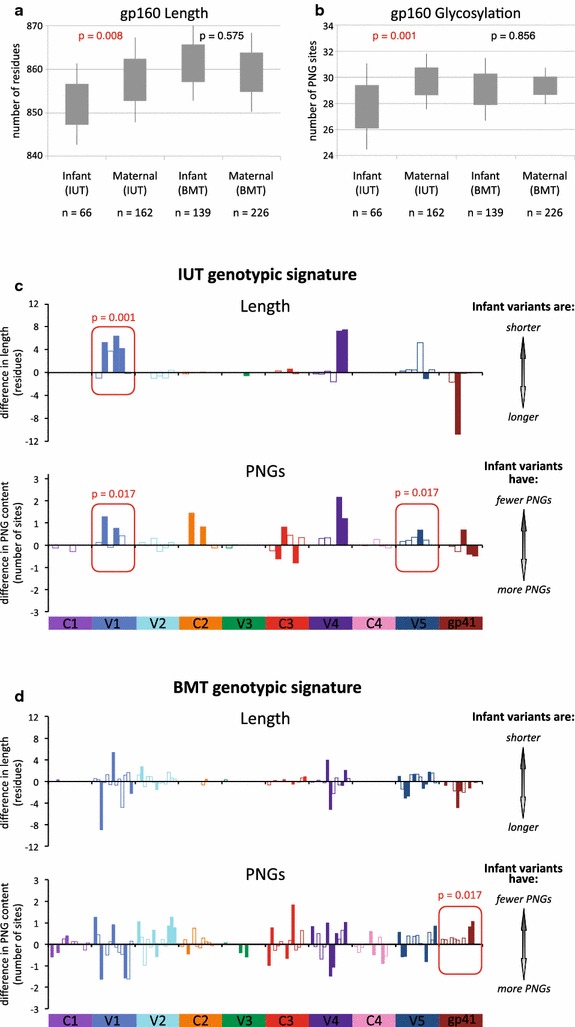
Genotypic signature for IUT and BMT groups. **a** Comparison of gp160 length (in number of amino acids) and **b** glycosylation level (in number of PNGs) for maternal and infant envelopes, segregated by route of transmission. The number of envelope sequences tested for each group (n) is given. **c**–**d** Comparison of infant and maternal envelope sequence length and PNG content, stratified by transmission mode and by gp120 variable domains (V), constant domains (C), and the gp41 subunit. *Each bar* along the horizontal axis represents an individual transmission pair, which is solid if comparison between the individual mother–infant pair is significant (p < 0.05) and outlined if not significant*. The y-axis represents either the difference in sequence length or the difference in number of PNG sites found between infant and maternal envelope clones, computed as [mean maternal isolate length (or PNG content)—the mean infant isolate length (or PNG content)] for each pair. p values were calculated using GEE for each individual domain, and domains that differed significantly for corresponding maternal and infant isolates are boxed in *red*. The graph in **c** shows differences in sequence length and PNG level for in utero transmission pairs, and **d** shows differences in length and PNG level for breast milk transmission pairs. *Wilcoxon rank-sum tests were used to compare sequence length or PNG content found in maternal and infant isolates from a given transmission pair, with resulting p values being corrected for multiple comparisons using the method of Benjamini and Hochberg [105]

In contrast to the overall comparisons between maternal and infants viruses described above, when stratified by transmission route, in utero transmission selected for gp160 variants that were shorter (mean length of 852.0 residues in infant variants and 857.6 in maternal variants; p = 0.008) and encoded fewer PNGs (mean of 27.8 sites in infant variants and 29.7 in maternal variants; p = 0.001) (Fig. 4a, b). When such analyses were confined to the V1–V4 region for the IUT group, the infant envelope variants had shorter V1–V4 lengths than maternal variants (mean of 277.0 residues in infant strains and 282.7 residues in maternal strains; p = 0.001) and fewer PNGs (mean of 18.8 sites within V1–V4 in infant strains, and 20.7 sites in V1–V4 of maternal strains; p = 0.001). This observed decrease in V1–V4 length and number of PNGs is similar to that encountered in studies of heterosexual transmission of HIV-1 subtype C [5], but neither was detected in our breast milk transmission group.

Examination of each individual variable and constant domains (V1–V5 and C1–C5) indicated that infant variants within the IUT group had shorter V1 loops than those found in their maternal counterparts (mean V1 length of 20.1 residues in infant isolates and 24.1 residues in maternal isolates; p = 0.001). Strains from infected infants also had fewer V1 PNGs (mean of 2.4 sites in infant isolates and 3.0 sites in maternal isolates; p = 0.017) and fewer V5 PNGs (mean of 1.2 sites in infant isolates and 1.5 sites in maternal isolates; p = 0.017) (Fig. 4c). No differences in length or number of PNGs for gp160, V1–V4, or V1 regions were observed in the BMT group (Fig. 4d). Infant isolates from the BMT group had fewer PNG sites only within gp41 (mean of 3.9 PNGs in gp41 of infant strains and 4.3 PNGs in gp41 from maternal strains; p = 0.017), while no such difference in gp41 PNGs was observed in the IUT group.

#### Inhibitor IC_50_ comparison

**Fig. 5:**
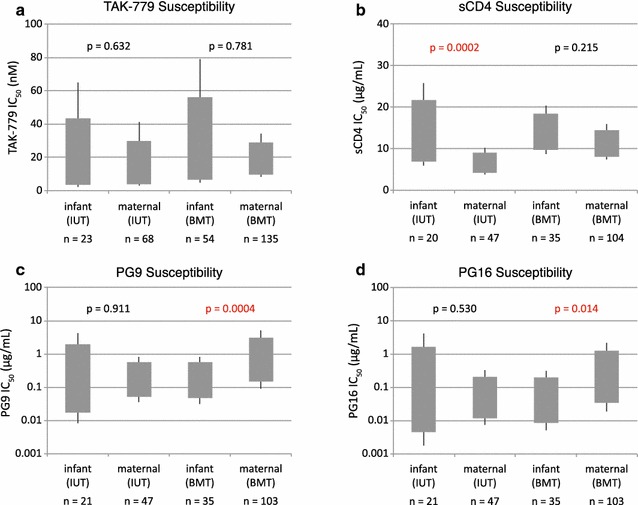
Phenotypic differences between variants transmitted in utero and through breast milk. Mean inhibitor IC_50_ values by subject and transmission mode were obtained through GEE calculations. The *grey box* indicates the 95% confidence interval and the *whiskers* indicate the 99% confidence interval. p values showing significant inhibitor IC_50_ differences for infant- and maternal-derived strains are indicated in *red*. Inhibitors tested were **a** TAK-779, **b** sCD4, **c** PG9, and **d** PG16. The number of envelope sequences tested for each group (n) is shown

In utero transmission failed to select infant-derived variants that differed significantly in bnAb sensitivity from maternal-derived variants, or in sensitivity to the inhibitors TAK-779 (Fig. 5) and T-20 (data not shown). However, GEE analysis of the mean sCD4 IC_50_’s showed that infant-derived variants from the IUT group were more resistant to sCD4 than corresponding maternal variants (mean sCD4 IC_50_ of 12.3 µg/mL for virus with infant-derived envelopes and 6.2 µg/mL for virus with maternal-derived envelopes; p = 0.0002) (Fig. 5). We further noted that when confining comparisons to within mother–infant pairs, the infant isolates showed higher median sCD4 IC_50_ values than those seen for corresponding maternal isolates in 5/6 IUT pairs (Additional file 4: Figure S4a). However, no statistically-significant difference in sCD4 IC_50_ for maternal and infant isolates within any given IUT mother–infant pair was confirmed (as determined by Wilcoxon rank-sum tests, followed by p value correction for multiple comparisons), perhaps owing to the relatively small number of envelopes being examined within each mother–infant pair. No difference in mean sCD4 sensitivity between infant- and maternal-derived isolates was observed in the BMT group, either in the aggregate or on a pair-by-pair basis (Additional file 4: Figure S4b).

Within the BMT group, infant variants were more susceptible than maternal variants to neutralization by bnAbs PG9 (mean IC_50_ of 0.16 µg/mL for virus with infant-derived envelopes and 0.68 µg/mL for virus with maternal-derived envelopes; p = 0.0004) and PG16 (mean IC_50_ of 0.04 µg/mL for virus with infant-derived envelopes and 0.21 µg/mL for virus with maternal-derived envelopes; p = 0.013), a difference that is not observed in the IUT group (Fig. 5). When comparing within BMT mother–infant pairs, the infant variants showed lower median PG9 and PG16 IC_50_ values than the corresponding maternal variants in 8/13 pairs (Additional file 4: Figure S4b). Using Wilcoxon rank-sum tests, no statistically significant differences in PG9 or PG16 IC_50_ values for the maternal and infant isolates within any of the BMT pairs were discerned, though this analysis may have been limited by the relatively low number of isolates analyzed within each BMT pair.

#### CD4 usage

In utero MTCT may depend upon initial infection of placental macrophages (e.g., Hofbauer cells), which have low surface densities of CD4 molecules [31, 63, 64]. To further explore the CD4 usage phenotype in our cohort, we investigated whether infant- and maternal-derived envelopes productively engage CD4 with differing efficiencies, and whether any difference in CD4 usage depends upon route of transmission. Towards this end, we used Affinofile cells [65] to test viral entry properties of 151 molecular envelope clones obtained from the 6 IUT and 11 of the 13 BMT pairs in our cohort (10 BMT pairs infected with HIV-1 subtype C and 1 with subtype G).

**Fig. 6:**
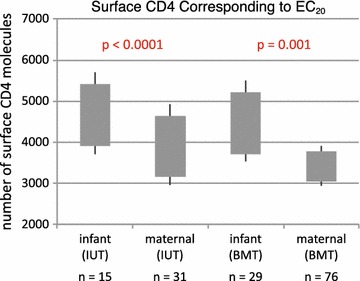
Surface CD4 molecules needed to produce 20% of maximum infectivity (EC_20_), by subject and transmission type. The number of envelope sequences tested for each group (n) is shown. The *grey box* indicates the 95% confidence interval and the *whiskers* indicate the 99% confidence interval

Affinofile cells can be induced to express varying levels of surface CD4 in a controllable fashion, allowing for standardized and reproducible infection of cells across a wide, physiologically-relevant range of CD4 surface densities. Affinofile cells were treated with ponasterone to maximally induce CCR5 expression, followed by addition of varying doses of doxycycline to yield controlled levels of CD4 expression. Infectivity curves were constructed by varying CD4 expression/cell over a 100-fold range, infecting cells with 2000 infectious units of virus pseudotyped with either infant or maternal envelope variants, and subsequently determining for each variant the number of CD4 molecules/cell (expressed as the number of α-CD4 antibody binding sites (ABS)/cell) required to achieve 20% of maximal entry (EC_20_). Analysis of EC_20_’s showed that infant-derived variants from the IUT group required more CD4 molecules than their maternal counterparts (mean EC_20_ of 4596 ABS/cell for virus with infant-derived envelopes and 3817 ABS/cell for virus with maternal-derived envelopes; p < 0.0001) (Fig. 6, Additional file 5: Figure S5). Pseudotyped virions from the BMT group showed similar behavior: infant variants in the BMT group required more CD4 molecules to achieve 20% of maximal entry than corresponding maternal strains (mean EC_20_ of 4409 ABS/cell for infant strains and 3387 ABS/cell for maternal variants; p = 0.001) (Fig. 6, Additional file 5: Figure S5). These results indicate that envelopes from the infant-derived strains in both IUT and BMT groups use CD4 less efficiently than corresponding maternal strains, as the infant strains require more CD4 molecules than their maternal counterparts to achieve comparable levels of target cell infection. Additionally, we evaluated CCR5 usage in a small subset of mother–infant pairs, 2 IUT and 4 BMT. Despite the small numbers, infant variants in the IUT group did require less CCR5 molecules for entry than corresponding maternal strains (mean EC_20_ of 411 α-CCR5 antibody binding sites (ABS)/cell) required to achieve 20% of maximal entry (EC_20_ABS/cell for infant strains and 493 ABS/cell for maternal variants; p < 0.001) (Additional file 6: Figures S6, Additional file 7: Figure S7). This difference was not seen in the BMT group (mean EC_20_ of 370 ABS/cell for infant strains and 553 ABS/cell for maternal variants; p = 0.153) nor when all 6 subjects were evaluated together (Additional file 6: Figures S6, Additional file 7: Figure S7).

#### *Dose*–*response curve analysis*

To further understand inhibition mechanisms for HIV-1 neutralization, and to ascertain functional differences in virions that are transmitted either in utero or through breast milk feeding, we fit inhibition curves of PG9 and sCD4 to the median effect model [66, 67]. In the median effect model, the slope parameter (m) provides a measure of allosteric cooperativity in inhibitor binding for multivalent targets, such as the HIV Env trimer. Slopes greater than 1 (m > 1) indicate positive cooperativity, where the binding of one inhibitor molecule to an Env spike allosterically increases the affinity of other binding sites on the trimeric Env. This favors the binding of additional inhibitor molecules and results in a steep dose-dependent rise in inhibitor activity and a high slope. Slopes less than 1 (m < 1) indicate negative cooperativity, where the inhibitor allosterically decreases the affinity of other binding sites, resulting in a shallow rise in inhibition and a low slope. Slopes equal to 1 (m = 1) indicate non-cooperativity, where an inhibitor has no allosteric effects on the Env structure.

**Fig. 7:**
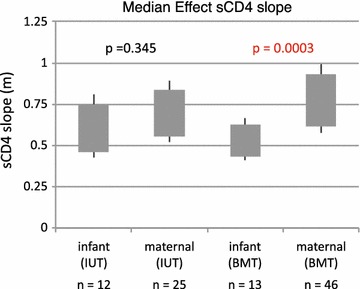
Comparison of median effect sCD4 slopes by subject and transmission mode. The number of envelope sequences tested for each group (n) is shown

Mean slope parameters obtained for PG9 against infant-derived and maternal-derived virions were both <1 and not significantly different from one another, either within the IUT or within the BMT groups (Additional file 8: Figure S8). Mean slope parameters for sCD4 inhibition of infant and maternal variants were also <1, and not significantly different within IUT group (mean slope of 0.59 for infant isolates and 0.68 for maternal isolates within the IUT group; p = 0.345) (Fig. 7). However, the sCD4 slopes for infant isolates in the BMT group were significantly lower than their maternal counterparts (mean slope of 0.52 for infant isolates and 0.76 for maternal isolates within the BMT group; p = 0.0003) (Additional file 9: Figure S9). Slopes that are <1, as seen in these sCD4 inhibition experiments, suggest that binding of an initial sCD4 molecule discourages binding of additional sCD4 molecules to the trimeric HIV-1 spike complex. Such behavior is characteristic of sCD4 inhibition for infant and maternal variants in both IUT and BMT groups in this cohort, and this negative cooperativity becomes more pronounced in the infant isolates from the BMT group relative to their maternal counterparts.

## Discussion

**Fig. 8:**
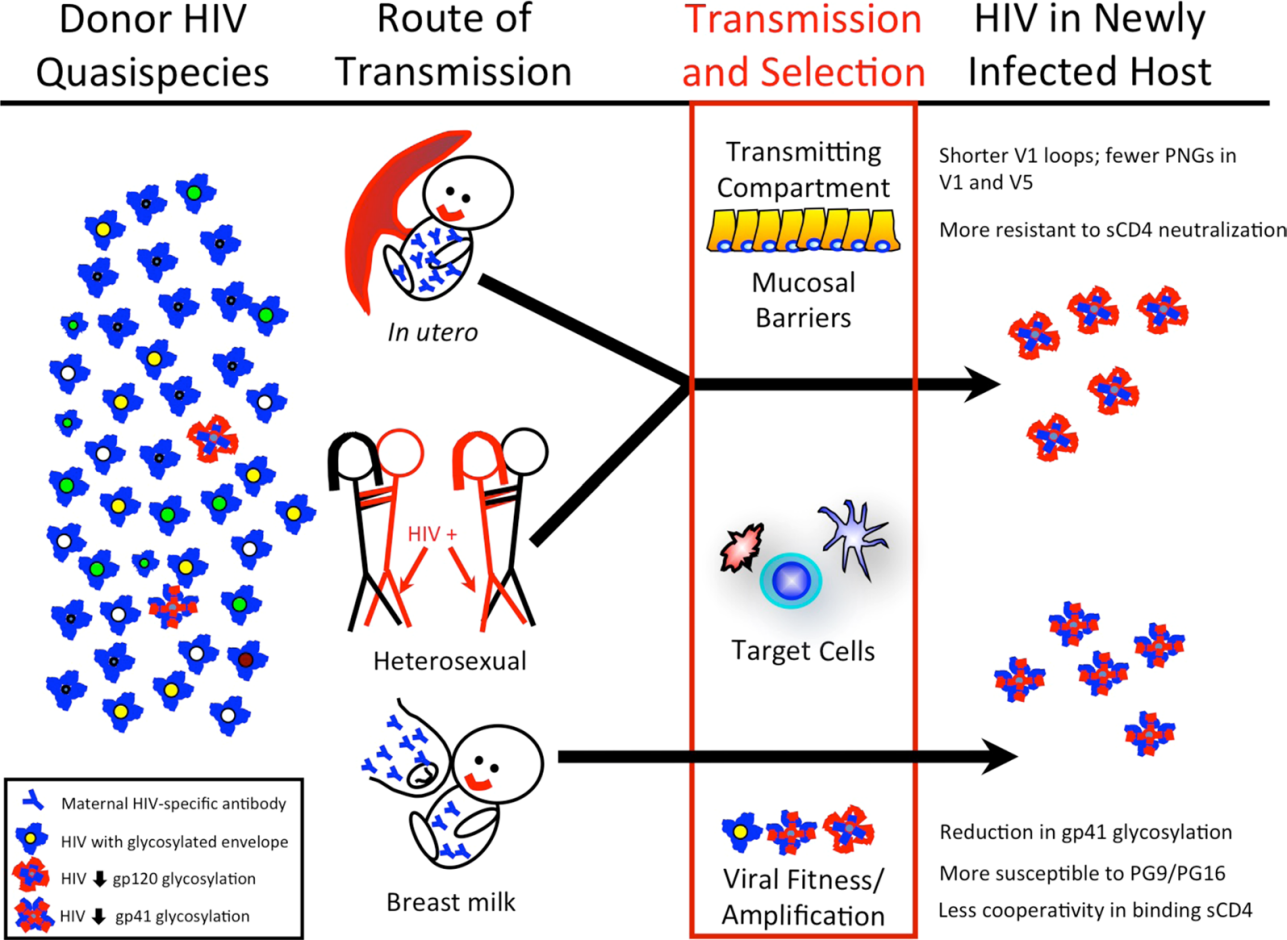
Genotypic and phenotypic features of HIV-1 isolates acquired in utero are distinct from those acquired through breastfeeding. On the *far left*, the pool of viral variants present in the chronically-infected donor are represented by differently-colored virion cores, with heavily-glycosylated envelopes coated in *blue* and those with less glycosylation coated in *red*. Strong selection during or following transmission (*red box*, *middle*) results in either a single or a very small number of variants that ultimately establish acute infection in the new host (*right*). Genotypic and phenotypic signatures by route of MTCT are as shown. For subtype C variants, heterosexual transmission bears similarity to transmission in utero, as both transmission modes select for isolates with shorter and less-glycosylated variable regions. MTCT of HIV-1 takes place in the presence of HIV-specific maternal antibody, while heterosexual transmission does not

Our detailed investigation of viral isolates obtained from maternal–infant transmission pairs revealed that genotypic and phenotypic features of HIV-1 envelopes acquired in utero (transplacental transmission) can be clearly distinguished from those acquired from breast milk (mucosal transmission). In utero transmission selects for HIV-1 subtype C infant isolates with shorter, less-glycosylated envelopes: specifically, the V1 domain of in utero-acquired envelope sequences is shorter, and the V1 and V5 domains contain fewer PNGs than corresponding maternal isolates. These observations suggest that envelopes with more compact V1 domains and less-glycosylated V1 and V5 domains impart a selective advantage to virions in establishing in utero infections. A recent study shows that HIV-1 subtype C isolates with compact V1/V2 regions have enhanced fusion efficiency and envelope incorporation into virions [68], and therefore the shorter V1 region within in utero-acquired strains in our present study may confer improved viral fusion kinetics and envelope uptake, potentially a basis for selection during intrauterine transmission. In contrast, breast milk transmission selects for HIV-1 variants that do not differ in overall length or glycosylation from maternal variants, but have fewer PNG sites in gp41, and are more sensitive to neutralization by the bnAbs PG9 and PG16. These particular genotypic and phenotypic signatures are unique to *either* intrauterine *or* breast milk transmission, but not both (Fig. 8).

Placental macrophages represent a population of HIV-susceptible fetal cells in close proximity to the maternal circulatory system, and express relatively low amounts of CD4 [63]. Previous work suggests that a higher affinity for CD4 correlates with increased susceptibility to sCD4 [69]. We therefore hypothesized that infection of placental macrophages would require variants with high CD4 affinity to scavenge the relatively scarce amounts of available CD4, and anticipated that envelope isolates from in utero-infected infants would thus be more sensitive to sCD4 inhibition. Contrary to our expectation, our data indicate that infant strains that emerged following in utero transmission display weaker interactions with the CD4 receptor and a lower binding affinity than maternal strain counterparts. Infection assays with TZM-bl cells show that infant envelope variants from the IUT group have an approximately twofold higher mean sCD4 IC_50_ value than that of corresponding maternal variants. These findings suggest that these infant-derived envelopes bind to cognate CD4 receptors on the cell surface with lower affinity than those from paired maternal isolates. In addition, our experiments with Affinofile cells show that all infant isolates require more surface CD4 than their maternal counterparts to achieve comparable levels of target cell infection. These data indicate that the transmitted infant variants exhibit a decreased CD4 usage efficiency reflected by both a decreased affinity and a higher stoichiometric requirement for entry. Conceivably, despite having lower CD4 affinity these infant envelope variants may have high CD4 avidity to achieve tight CD4 binding in this setting, exploiting interactions of multiple trimeric spike protein subunits with clusters of CD4 receptors in a contact zone (a so-called “entry claw”) [70, 71] to successfully attach to and infect target cells. Here, an elevated CD4 surface density promotes high HIV-1 envelope-CD4 avidity and ensuing infection. One additional possibility that is consistent with our sCD4 data is that the target fetal cells initially infected during in utero transmission actually express a relatively higher amount of CD4, which may result if a transient inflammatory process occurs during gestation, resulting in elevated CD4 expression and facilitating MTCT despite transmitted isolates having a relatively low CD4 affinity.

Alternatively, in utero-transmitted isolates with weakened CD4 affinity might actually possess a reduced reliance upon CD4, perhaps by assuming envelope conformations that are predisposed to immediate CCR5 engagement. In an infection model that requires little CD4, a reduced CD4 affinity would pose no great fitness penalty: conceivably, any additional surface CD4 density beyond a certain threshold level could help promote infection, but this excess CD4 would not be strictly required. Our dose–response curve analysis of the sCD4 inhibition data using the median effect framework [67] is consistent with this later interpretation. The mean sCD4 slope parameter for in utero-transmitted infant variants, though not significantly different from that of paired maternal variants, is <1 (mean slope of ~0.6). This low slope parameter suggests that initial envelope trimer engagement of a CD4 molecule discourages subsequent binding of additional CD4 molecules (i.e., negative cooperativity), perhaps by the envelope adopting a conformation that occludes or disturbs the remaining CD4 binding sites on the trimer. Such a conformation might confer a selective advantage by better exposing the CCR5 co-receptor binding site, providing easier access for CCR5 co-receptor and relying less upon the usual CD4 binding event(s) to engage CCR5 and subsequently trigger viral fusion. The low slope parameter observed in our study might also imply slower envelope activation kinetics, which would provide additional time to assemble an entry-competent complex with co-receptor following an initial CD4 binding event.

While most HIV-1 isolates are CD4-dependent, some variants with low or no requirement for CD4 have been described (reviewed in Ref. [72]). Brain-derived, macrophage-tropic HIV-1 envelope variants with low CD4 requirements and increased fusogenicity have been isolated [73], and macrophage tropism of simian immunodeficiency virus (SIV) is associated with CD4 independence [74, 75] and reduced CD4 affinity [76]. In addition, data from other Affinofile cell experiments suggest that HIV-1 macrophage tropism requires little CD4 (but is not completely CD4-independent) [77]. Given our data and observations from studies described above, we surmise that selective pressures during in utero transmission yield infant isolates with low CD4 requirements for productive infection, and that are well-poised to infect placental macrophages (e.g., Hofbauer cells) that have lower cell surface CD4 densities, perhaps through exploiting higher CD4 avidity, direct co-receptor binding, and/or having enhanced fusogenic properties.

Our studies provide other insights into factors that select the HIV variants that establish infection. Replication of viral lineages that are genetically distinct from those circulating in the blood can be found within the central nervous system [78, 79], in the genital tract [80], and within the placenta [31]. Conversely, we and others have previously shown that virus found in maternal blood and in breast milk and are not genetically distinct from one another [81–84], suggesting that the observed genetic restriction during BMT must occur within recipient infant tissues, perhaps involving specific cellular factors.

HIV variants from infants infected in utero in our study were similar to those described in heterosexual transmission of HIV-1 subtypes A and C [5, 23], i.e., having shorter variable loops with fewer PNGs than those found in the transmitting partner. The finding that variants that traverse the placenta have similar genotypic characteristics to those that breach the male and female genital tracts suggests that these different anatomical compartments impose common selective pressures that favor (in each setting) transmission of virions with more compact envelope variable regions having fewer PNGs (Fig. 8). Although the origin of such selective pressures is currently unknown, candidates under consideration include cellular lectins (reviewed in Ref. [85]), whose presence could potentially favor transmission of under-glycosylated envelope isolates that can escape the innate immune system, or the cellular integrin α_4_β_7_, which may facilitate mucosal transmission through strong interactions with less-glycosylated V1/V2 regions [86]. Finally, a strong requirement for virions with elevated envelope incorporation and efficient viral fusion properties during heterosexual and in utero transmission might lead to the selection of isolates having compact V1/V2 regions, which have previously been shown to display this phenotype [68].

Unlike other forms of HIV transmission, MTCT occurs in the presence of HIV specific antibody. At birth, IgG antibody levels in the infant are equal or greater than those in the mother, but rapidly decline during the first few months of life [42, 87]. Although some studies indicate that founder variants in infants have escaped maternal neutralizing antibody [30], other studies have shown that neither breadth nor potency of passively acquired maternal antibody protect against breast milk transmission [88] and that maternal antibody does not strongly influence founder selection [32]. Nevertheless, the search for broadly neutralizing antibodies (bnAbs) that can serve either as a basis for vaccine design or as passively-administered therapeutics remains an active area of research [89]. The increased susceptibility to PG9 and PG16 of subtype C and G isolates from infants infected through breast milk in our study is consistent with observations from a previous report, which also showed that variants from infants infected through breast milk are sensitive to PG9 and PG16 [33]. In addition, subtype CRF01_AE isolates from infants infected perinatally also display increased PG9 and PG16 sensitivity over corresponding maternal strains [90]. Collectively, these findings support the exploration of bnAbs such as PG9 and PG16 in MTCT clinical trials.

## Conclusions

In summary, our data indicate that different MTCT routes select genotypically and phenotypically distinct viral variants. These findings may have implications for both HIV pathophysiology and the development of successful MTCT intervention strategies. Past studies suggest that infants infected perinatally experience more rapid disease progression than those infected later by breastfeeding, with the accelerated pathogenesis being attributed to differences in immunologic maturity at the time of infection, and/or to detrimental effects of HIV infection upon infant growth and development [91, 92]. Alternatively, our data suggest that characteristic viral isolates for each transmission setting might infiltrate target tissues through unique mechanisms that may contribute to the observed differences in disease progression. Further comparison analyses of the HIV strains that are selected by different MTCT routes could elucidate these mechanisms, as well as lead to new therapeutic strategies or vaccine designs aimed at blocking vertical transmission in a mode-specific fashion.

## Methods

### Subjects

We selected 22 women who were enrolled in the Zambia Exclusive Breastfeeding Study (ZEBS) [44] and who were HIV-positive and pregnant at the time of enrollment. The mothers and infants in our study population received a single-dose of nevirapine peripartum, as per Zambian government guidelines at the time of study enrollment. Infants were also given cotrimoxazole prophylaxis from 6 weeks to 12 months of age [44]. The mode of viral transmission for each mother–infant pair was determined by the timing of the first HIV-1 PCR-positive test for the infant: infants who were showed a PCR-positive test at birth were deemed to have acquired virus in utero (n = 6), while those infants who were PCR-negative at birth and at 1 month of age but PCR-positive after 42 days were considered to be infected through breastfeeding (n = 13). Infants whose HIV-1 PCR tests were negative at birth but positive after 1 month were considered to have acquired virus either intrapartum or early postpartum (n = 3).

### Envelope cloning

Peripheral blood and whole breast milk was collected at regular intervals starting at study enrollment (for maternal blood), and extending to 24 months post-delivery. Peripheral blood mononuclear cells (PBMCs) were separated by Ficoll–Hypaque centrifugation (MP Biomedicals) and preserved with RNAlater (Applied Biosystems) or DNAzol (Invitrogen), followed by freezing of both cells and cell-free plasma. Cell and supernatant/lipid fractions from breast milk were separated by centrifugation and then frozen.

Viral RNA copy numbers were determined from maternal plasma samples using the Roche Amplicor v1.5 standard or ultrasensitive methods (Roche Diagnostics). Full-length gp160 sequences were amplified and cloned from either reverse-transcribed plasma RNA or from DNA extracted from PBMCs or breast milk cells using nested PCR with Vif1/OFM19 (outer) and EnvA/EnvN (inner) primer pairs, as previously described [5, 93]. Multiple independent PCR reactions were performed at or near limiting dilution (i.e. 0.1–10 template copies per reaction) to favor amplification of a single viral envelope sequence. The *env* sequences from the 647 clones isolated in this cohort were obtained through bidirectional Sanger sequencing, and initially analyzed using Sequencher (Genecodes), MacVector (MacVector Inc), and the LANL website tools (http://www.hiv.lanl.gov/content/sequence/LOCATE/locate.html). Examination of *env* sequences proceeded as has been described previously [93]. In brief, all chromatograms from Sanger sequencing runs were visually inspected during assembly, and any chromatograms with dual peaks were excluded from further analysis. When multiple clones that resulted from a single PCR reaction were not identical, suggesting that more than one amplifiable template was present during PCR amplification, only one representative clone was selected for further analysis.

### Genetic analysis

Examination of *env* sequences proceeded much as has been described previously [93]. In brief, all chromatograms from Sanger sequencing runs were visually inspected during assembly, and any chromatograms with dual peaks were excluded from further analysis. All sequences were compared against the HIV-1 database using ViroBLAST [94] to rule out cross-contamination. Sequences were aligned in MUSCLE v3.7 [95] and refined manually using Geneious (Biomatters, Auckland). A maximum likelihood tree was calculated in PhyML v3.0 [96] using the online tool DIVEIN (http://indra.mullins.microbiol.washington.edu/DIVEIN/) (doi: 10.2144/000113370), which implements the evolutionary model GTR + I + G. The tree was rooted with the subtype B reference sequence HXB2 and represented using FigTree version 1.3.1 (http://tree.bio.ed.ac.uk/software/figtree). Potential N-linked glycosylation sites (PNGs) were identified using the *N*-Glycosite tool on the LANL website (http://www.hiv.lanl.gov/content/sequence/GLYCOSITE/glycosite.html) [97].

The number of founder variants establishing infection was inferred by detailed assessment of the infant virus populations, including the number of phylogenetically informative mutation sites (i.e., specific mutations found in more than one sequence, or “InSites”) as determined by DIVEIN; the number of observed sublineages (multiple sequences sharing the same InSites) and the number of mutations defining the sublineage; whether insertions or deletions (InDels) were detected and shared across different sequences; whether probable recombination was observed; and the number of maternal sequences found to represent the most recent common ancestor (MRCA) associated with transmission. The amount of time between PCR-based identification of infant infection and virus population sampling was also considered in making this determination (Additional file 10: Table S1; Additional file 2: Figures S2, Additional file 3: Figure S3).

### Cells and reagents

293T retroviral packaging cells were obtained from either the American Type Culture Collection (ATCC) or from Clontech (Lenti-X 293T cells), and TZM-bl cells were obtained through the National Institutes of Health (NIH) AIDS Reagent Program (catalog #8129), courtesy of Dr. John C. Kappes, Dr. Xiaoyun Wu and Tranzyme Inc. TZM-bl is a HeLa clone that constitutively expresses high surface levels of CD4, CCR5, and CXCR4 and contains both luciferase and β-galactosidase reporter genes under control of the HIV-1 LTR promoter [98–101]. TZM-bl and 293T packaging cells were maintained in Dulbecco’s modified Eagle’s medium (DMEM) (Fisher Scientific) supplemented with 10% fetal bovine serum (Gemini Bio-products), 100 U/mL penicillin-100 µg/µL streptomycin (Gibco), and 2 mM l-glutamine (Gibco). GHOST-R5 (expressing CCR5) and GHOST-X4 (expressing CXCR4) cells were also obtained from the NIH AIDS Reagent Program (catalog #3944 and #3685, respectively), courtesy of Dr. Vineet N. KewalRamani and Dr. Dan R. Littman, and were maintained in DMEM with 4.5 g/L glucose (Fisher Scientific), 10% FBS, 500 µg/mL G418 (Invitrogen), 100 µg/mL hygromycin (Invitrogen), and 1 µg/mL puromycin (Sigma-Aldrich). Affinofile cells have been previously described [65]; briefly, these cells contain independently inducible CD4 and CCR5 expression constructs under the regulatory control of tetracycline and ponasterone, respectively, as well as an HIV-promoter-driven, secreted Gaussia luciferase reporter. Affinofile cells were maintained in Affinofile media (AfM) containing Advanced DMEM (Gibco, Carlsbad, CA) supplemented with 2% dialyzed FBS (Hyclone, Waltham, MA), 2 mM l-glutamine (HiMedia), 100 U/mL penicillin G/100 µg/µL streptomycin (Mediatech), and 50 µg/mL blasticidin (Mediatech). QuantiBRITE™ beads for flow cytometry experiments with Affinofile cells were purchased from BD Biosciences (catalog #340495; Franklin Lakes, NJ). Phycoerythrin (PE)-conjugated mouse anti-human CD4 (clone RPA-T4, catalog #555347), anti-human CCR5 (clone 2D7, catalog #555993), and isotype control (clone MOPC-21, catalog #555749) monoclonal antibodies (mAbs) with a 1:1 antibody:PE ratio were also purchased from BD Biosciences.

The following drugs and antibodies were obtained through the NIH AIDS Reagent Program: HIV-1 gp41 mAbs 4E10 (catalog #10091) and 2F5 (catalog #1475), courtesy of Dr. Hermann Katinger [48–51]; HIV-1 gp120 mAb IgG1 b12 (catalog #2640) from Drs. Dennis Burton and Carlos Barbas [54–57]; TAK-779 from Takeda Chemical Industries, Ltd. (catalog #4983) [60, 61]; T-20 from Roche (catalog #9845); and sCD4-183 (catalog #7356) from Pharmacia, Inc. [59] The mAbs 4E10 and IgG1 b12 were also purchased directly from Polymun Scientific. The bnAbs PG9 and PG16 were kindly provided through the International AIDS Vaccine Initiative (IAVI), courtesy of Dr. Dennis Burton and the Protocol G team.

### Pseudovirus production

Pseudovirus was produced by co-transfection of 293T cells with a plasmid containing a cloned maternal or infant *env* sequence and the pSG3∆Env backbone (obtained through the NIH AIDS Reagent Program; catalog #11051) using Fugene 6 (Roche Applied Science). The culture medium was clarified by centrifugation at 48–72 h post-transfection, and then aliquoted and stored at −80 °C. One aliquot was thawed and titered on TZM-bl cells. The percentage of functional clones relative to the total number of clones amplified varied from 6 to 90% among the transmission pairs.

### Neutralization assays

Infectivity assays were performed in a 96-well plate format using TZM-bl cells as previously described [93]. Fifty percent inhibitory concentrations (IC_50_’s) for each tested inhibitor were interpolated from data points immediately above and below the 50% infectivity level in log-linear plots of dose–response curves using Excel (Microsoft). Each infectivity data point in the dose response curve represented the mean of at least two separate tests of a given inhibitor concentration and showed less then 30% variation among these tests.

### Affinofile assay

On Day 0, Affinofile cells were trypsinized (TrypLE; Lifetech, Carlsbad, CA), washed once with AfM, counted, and diluted to 200,000 cells/mL prior to induction. An aliquot of uninduced cells treated with carrier controls (ethanol and deionized water) was replated, and the remaining cells were treated with 2 µM ponasterone (Invitrogen/Life Technologies) to induce high levels of CCR5 expression. CCR5-induced cells were then split into six pools for treatment with doses of doxycycline (Calbiochem, Billerica, MA) ranging between 0.1 and 1.6 ng/mL to induce CD4 expression. Doxycycline-induced cells were plated at 20,000 cells/well in 96-well plates, with a small remainder reserved for culture in a T-25 flask for subsequent flow cytometry analysis.

On Day 1, media was aspirated from all wells and 2000 IU virus was added to duplicate wells of a 96-well plate after being diluted in AfM without diethylaminoethyl (DEAE)-dextran to a total volume of 200 µL, which yielded 10,000–20,000 relative light units (RLUs) per well after a 48 h incubation. AfM without virus was added to uninfected cell control wells. The induced and uninduced Affinofile cells were trypsinized in T25 flasks and washed. Receptor expression levels were subsequently measured by quantitative fluorescence-activated flow cytometry (qFACS) using PE-conjugated α-CD4 and α-CCR5 mAbs, PE-conjugated QuantiBRITE™ beads, and a LSR II flow cytometer (Becton–Dickinson). A standard curve generated from measurements with the QuantiBRITE™ beads having known PE levels allowed calculation of cell surface concentrations of CD4 (expressed as antibody binding sites (ABS)/cell), taking into account that the α-CD4 mAbs used in Affinofile experiments have a 1:1 PE:antibody molar ratio.

On Day 3, 30 µL of luciferase-containing supernatant was transferred from wells containing infected or mock-infected cells to a 96-well white opaque plate and read on a Lumistar Optima instrument (BMG Labtech, Cary, NC) using a Gaussia-luciferase kit (New England Biolabs, Ipswich, MA) per manufacturer’s instructions. RLU values were compared between duplicate wells and any pairs with >25% variation were discarded as invalid. Data from an entire sample were discarded if the 1.6 ng/mL doxycycline wells (high control) were either invalid due to unacceptable variance, or less than threefold higher RLU readout than the cell control well values. RLU values for each pair of wells were averaged and the mean cell-control value subtracted before normalization (as a percentage) to the 1.6 ng/mL doxycycline RLU value for that sample. Percent infectivity values were then pooled across a minimum of three independent experiments for each sample and plotted in Excel (Microsoft, Redmond, WA) against the absolute number of CD4 molecules/cell (as determined through comparison to the QuantiBRITE™-derived standard curve) to construct a single infectivity curve for each isolate across a ~100-fold range of CD4 expression levels. Each infectivity curve was used to calculate the number of CD4 molecules required to achieve 20% of maximum entry (EC_20_) for a given variant. The 20% value was chosen based on an early analysis of control viruses, before the bulk of the data were analyzed. These assay parameters consistently resulted in detectable, low-level viral entry and good assay-to-assay reproducibility.

CCR5 infectivity curves were generated using the same protocol, except CD4 was induced to saturating levels with 3 ng/mL doxycycline and CCR5 levels were adjusted using a ponasterone concentration range of 3–0.05 µM, giving a 100-fold range of CCR5 surface density (approximately 100–10,000 CCR5 ABS/cell).

### Median effect analysis

The median effect framework provides characterization of viral inhibitors through a logarithmic transformation of dose response curves from infection assays [66, 67], as described in Eq. 1:

1 $$\log \left( {\frac{{f_{a} }}{{1 - f_{a} }}} \right) = m\log \left( D \right) - m\log \left( {D_{m} } \right)$$

where *f*_*a*_ is the fraction of affected virus, *D* is the inhibitor concentration, *D*_*m*_ represents the inhibitor concentration that causes 50% of the maximum inhibitory effect (equivalently, the inhibitor IC_50_), and *m* is a slope parameter that is analogous to the Hill coefficient [102] and provides a measure of the cooperativity of the system. The median effect model was fit to viral inhibition data using linear regression with in-house perl scripts, providing slope and IC_50_ values of a given inhibitor for each tested maternal and infant isolate. In all cases, median effect fits were determined from the average of two experimental replicates for each neutralization curve, and isolates for which the squared correlation coefficient (R^2^) was 0.9 or better were selected for further analysis. Those isolates that could not be neutralized by 50% within the range of inhibitor concentrations used in viral infection assays were not analyzed further.

### Statistical analysis

We used GEE modeling to compare mean values of genotypic and phenotypic features between mother and infant sequences. GEE is a parametric method that takes into account differences in the number of isolates tested per patient, as well as the relatedness of linked maternal and infant isolates. All inhibitor IC_50_ datasets were log_10_-transformed prior to GEE analysis to yield a normal distribution, and an exchangeable correlation matrix was used for GEE calculations. Data analysis was conducted using SAS 9.1 (Cary, NC, USA) and R [103]. Diversity comparisons were done using the method of Gilbert et al. [104], and proportion of putative multi-variant transmissions were compared using Fisher’s exact test. All reported p values are based on GEE testing unless otherwise specified, and were adjusted for multiple comparisons using the method of Benjamini and Hochberg to control for the false discovery rate [105].

## Accession numbers

 Nucleotide sequences associated with this manuscript have been submitted to GenBank with accession numbers: GU939049–GU939104, GU939106, GU939107, GU939124–GU939142, HM036739–HM036745, HM036747–HM036749, HM036751–HM036789, HM036791, HM036793, HM036794, HM036797–HM036800, HM036802–HM036831, HM036983–HM037012, HM037015–HM037021, HM037023–HM037037 and KY229251–KY229682. Alignments are available at: https://mullinslab.microbiol.washington.edu/publications/nakamura_2016/.

## Additional files

**Additional file 1: Figure S1.**Domains of the HIV-1 envelope proteins. SP, signal peptide; C1–C5, conserved domains; V1–V5, variable domains; ECD, gp41 extracellular domain; TM, gp41 transmembrane domain; CD, gp41 cytoplasmic domain.

**Additional file 2: Figure S2.**Pairwise comparisons of genetic diversity within infant founder virus populations. All pairwise comparisons are shown scaled to the same genetic divergence in each panel on the X-axis, with bin intervals of 0.002, but with differentially scaled Y axes that reflect the variable number of sequences obtained from each infant.

**Additional file 3: Figure S3.**Phylogenetically informative mutation sites and assessment of single versus multiple virus founders in infants. (a) Number of InSites in the infant virus founder populations and the assessment of single versus multiple founders. Note that tally of these sites were only one of several criteria used to assess funder numbers (see Additional file 10: Table S1 for full list of criteria). (b, c) Phylogenetic trees and alignment of phylogenetically informative sites for sequences from the two infants in panel (a) with 6 such sites. These were the only two infants for which the data across all criteria were not unambiguous. While both had evidence of multiple variants, the presence of two founders defined by GGC versus AAA (the latter in the lower infant clade) was clearer in B19, whereas the multiple variants in B16 could have resulted from immune escape when sampled at 88 days of virus positivity.

**Additional file 4: Figure S4.**sCD4, TAK-779, PG9, and PG16 IC_50_ data for maternal- and infant-derived envelopes, presented on a pair-by-pair basis. (a) the IUT group and (b) the BMT group. Median IC_50_ values are indicated by a horizontal bar. Wilcoxon rank-sum tests were used to compare IC_50_ values obtained from maternal and infant isolates from a given maternal–infant pair, with resulting p values being corrected for multiple comparisons using the method of Benjamini and Hochberg [105]. Any p values showing a significant difference between IC_50_’s from the maternal- and infant-derived variants (p ≤ 0.05) are indicated in red.

**Additional file 5: Figure S5.**Representative CD4 infectivity curves using Affinofile cells for IUT (*top*) and BMT (*bottom*) maternal–infant pairs. Affinofile cells were induced to generate a 100-fold range of CD4 surface density (ABS/cell) and infected with 2000 IU pseudotyped virus. Percent infection was measured as the percent luciferase relative to infected and maximally induced Affinofile cells. Data shown are representative curves among 3–4 experimental replicates.

**Additional file 6: Figure S6.**Surface CCR5 molecules needed to produce 20% of maximum infectivity (EC_20_), by subject and transmission type. The number of envelope sequences tested for each group (n) is shown. The grey box indicates the 95% confidence interval and the whiskers indicate the 99% confidence interval.

**Additional file 7: Figure S7.**CCR5 infectivity curves using Affinofile cells for IUT (top) and BMT (bottom) maternal–infant pairs. Affinofile cells were induced to generate a 100-fold range of CCR5 surface density (ABS/cell) and infected with 2000 IU pseudotyped virus. Percent infection was measured as the percent luciferase relative to infected and maximally induced Affinofile cells. Data shown are the average of three replicates.

**Additional file 8: Figure S8.**Representative PG9 neutralization curves for IUT (top) and BMT (bottom) maternal–infant pairs. Data shown are representative curves among 3–4 experimental replicates.

**Additional file 9: Figure S9.**Representative sCD4 neutralization curves for IUT (left) and BMT (right) maternal–infant pairs. Data shown are representative curves among 3–4 experimental replicates.

**Additional file 10: Table S1.**Identification of founder variants establishing infection. Infant infections that were inferred to be associated with single (S) or multiple (M) founder variants are shown, along with data used to make these determinations. Given the varying time between the first PCR-positive HIV test and virus population sampling in the infected infant, the wide range in the number of maternal and infant viral sequences sampled, and the unknown impact of early immune responses in selecting for mutations in the virus population, multiple criteria were considered when assessing whether transmission was associated with single or multiple founder variants. These criteria included (i) the number of phylogenetically informative mutation sites (InSites) computed by DIVEIN (doi: 10.2144/000113370), (ii) the minimum number of viral sublineages (multiple sequences sharing the same InSites), (iii) the estimated number of shared mutations defining these sublineages, (iv) whether insertions or deletions (InDels) were detected and shared across different sequences, (v) whether probable recombination was observed between sequences, and (vi) the number of maternal sequences found to represent the most recent common ancestor (MRCA) of the infant’s infection in phylogenetic trees. Multiple founders were inferred when the infant virus population emerged from more than a single MRCA in the mother, and/or when sublineages were detected that differed by 4 or more mutations (including InDels). Supporting these inferences were the detection of recombinants between sublineage sequences. The number of sublineages and mutations defining these sublineages were in some cases not precisely defined due to recombination. While in utero transmission showed a higher incidence of multiple founder virus outgrowth (67% in the IUT group versus 31% in the BMT group), our sampling was insufficient to determine a statistically-significant difference (p = 0.319, Fisher’s exact test).
