# Supplementary figures and images for: Breast milk and in utero transmission of HIV-1 select for envelope variants with unique molecular signatures
Source: Retrovirology. 2017 Jan 26;14:6. doi: 10.1186/s12977-017-0331-z (PMC5267468; doi:10.1186/s12977-017-0331-z)

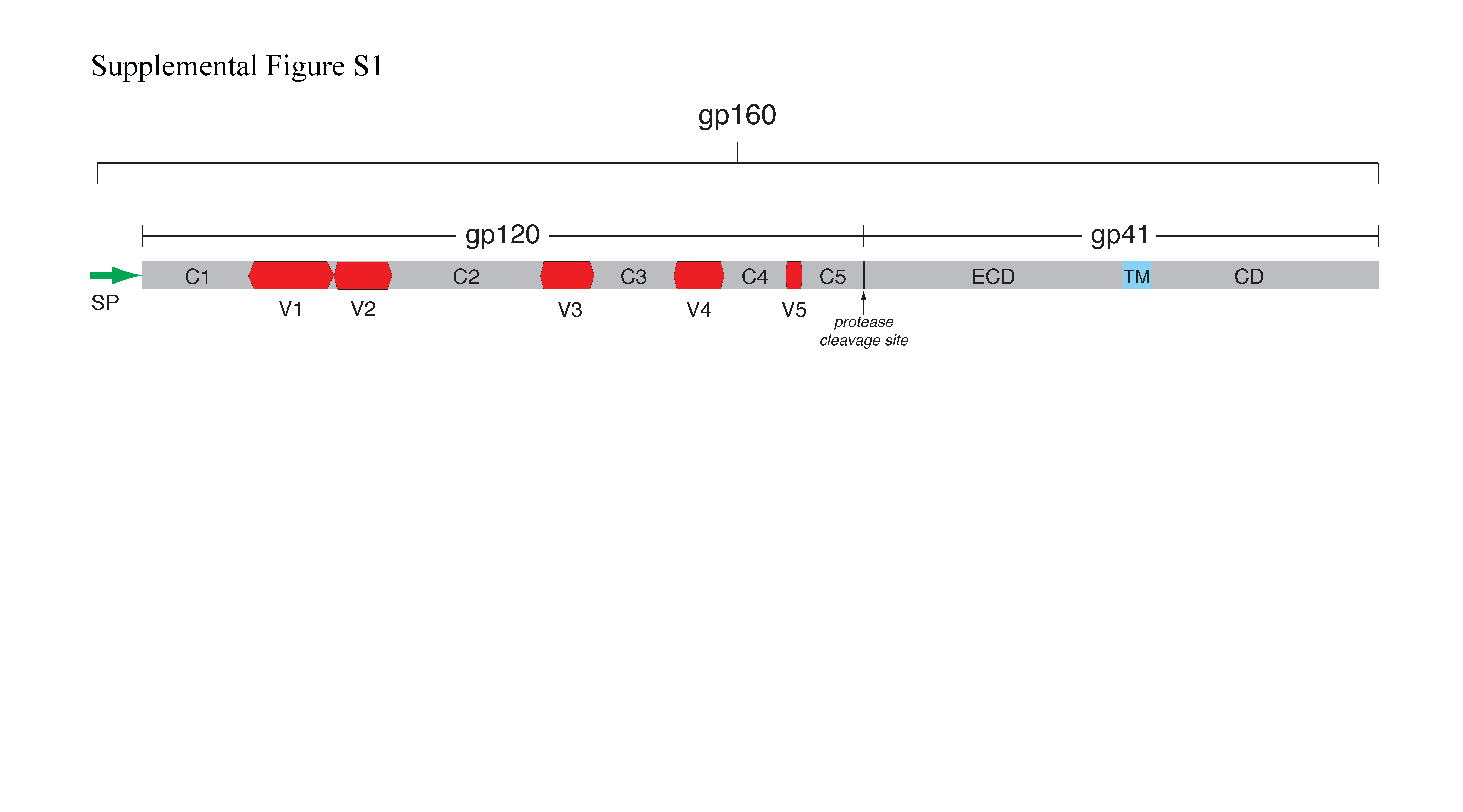

Supplement: Supplementary file 1 — Additional file 1: Figure S1.Domains of the HIV-1 envelope proteins. SP, signal peptide; C1–C5, conserved domains; V1–V5, variable domains; ECD, gp41 extracellular domain; TM, gp41 transmembrane domain; CD, gp41 cytoplasmic domain. [file 12977_2017_331_MOESM1_ESM.tiff]

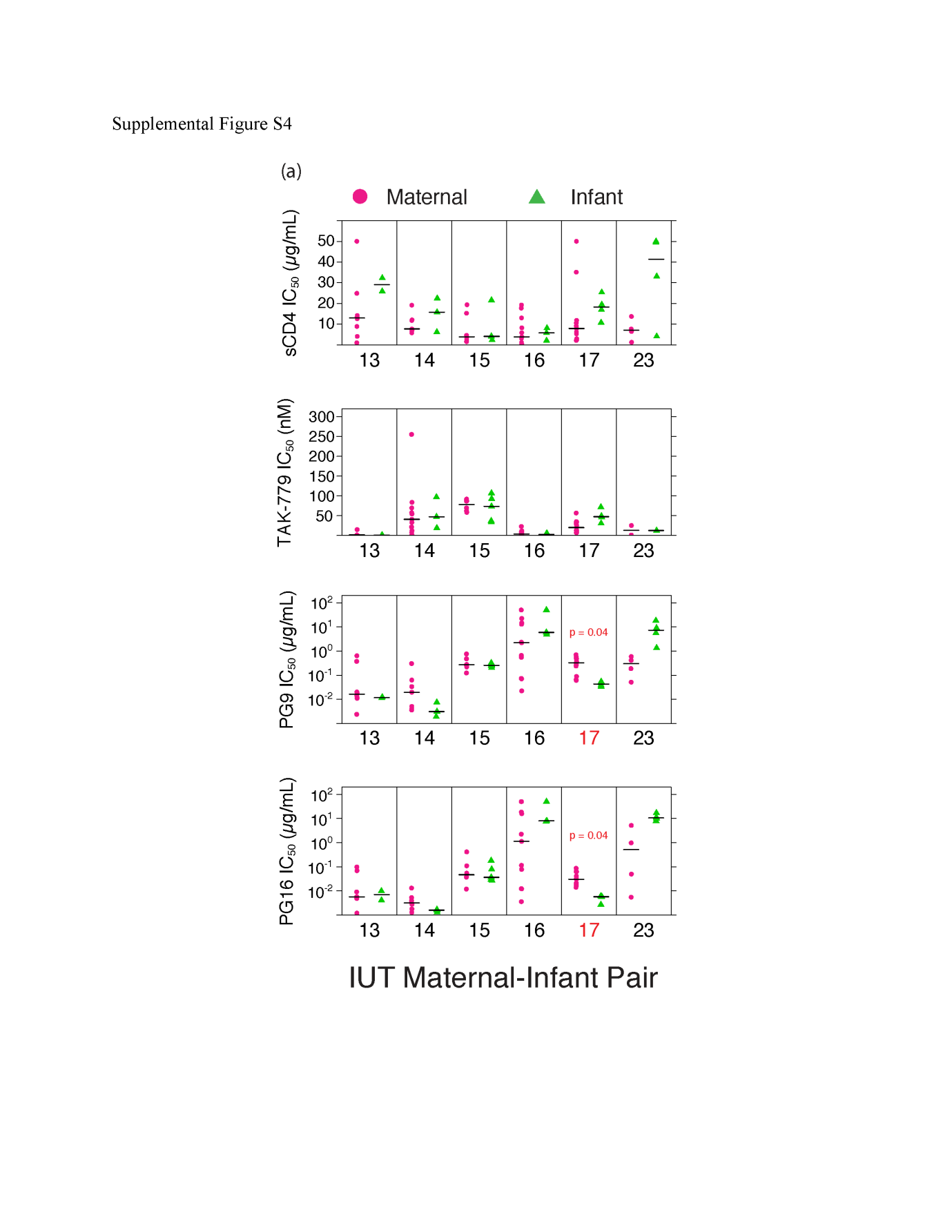

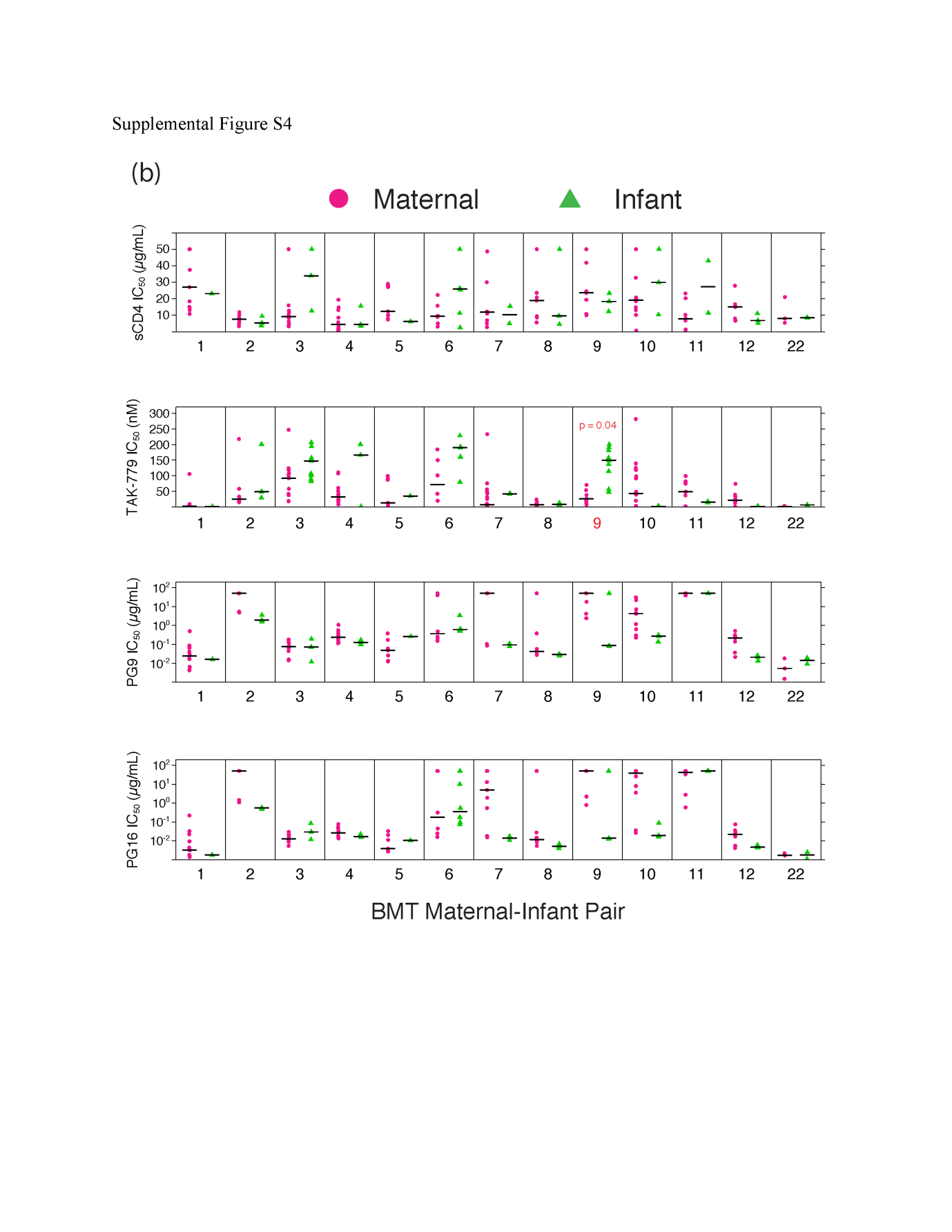

Supplement: Supplementary file 4 — Additional file 4: Figure S4.sCD4, TAK-779, PG9, and PG16 IC50 data for maternal- and infant-derived envelopes, presented on a pair-by-pair basis. (a) the IUT group and (b) the BMT group. Median IC50 values are indicated by a horizontal bar. Wilcoxon rank-sum tests were used to compare IC50 values obtained from maternal and infant isolates from a given maternal–infant pair, with resulting p values being corrected for multiple comparisons using the method of Benjamini and Hochberg [105]. Any p values showing a significant difference between IC50’s from the maternal- and infant-derived variants (p ≤ 0.05) are indicated in red. [file 12977_2017_331_MOESM4_ESM.docx]

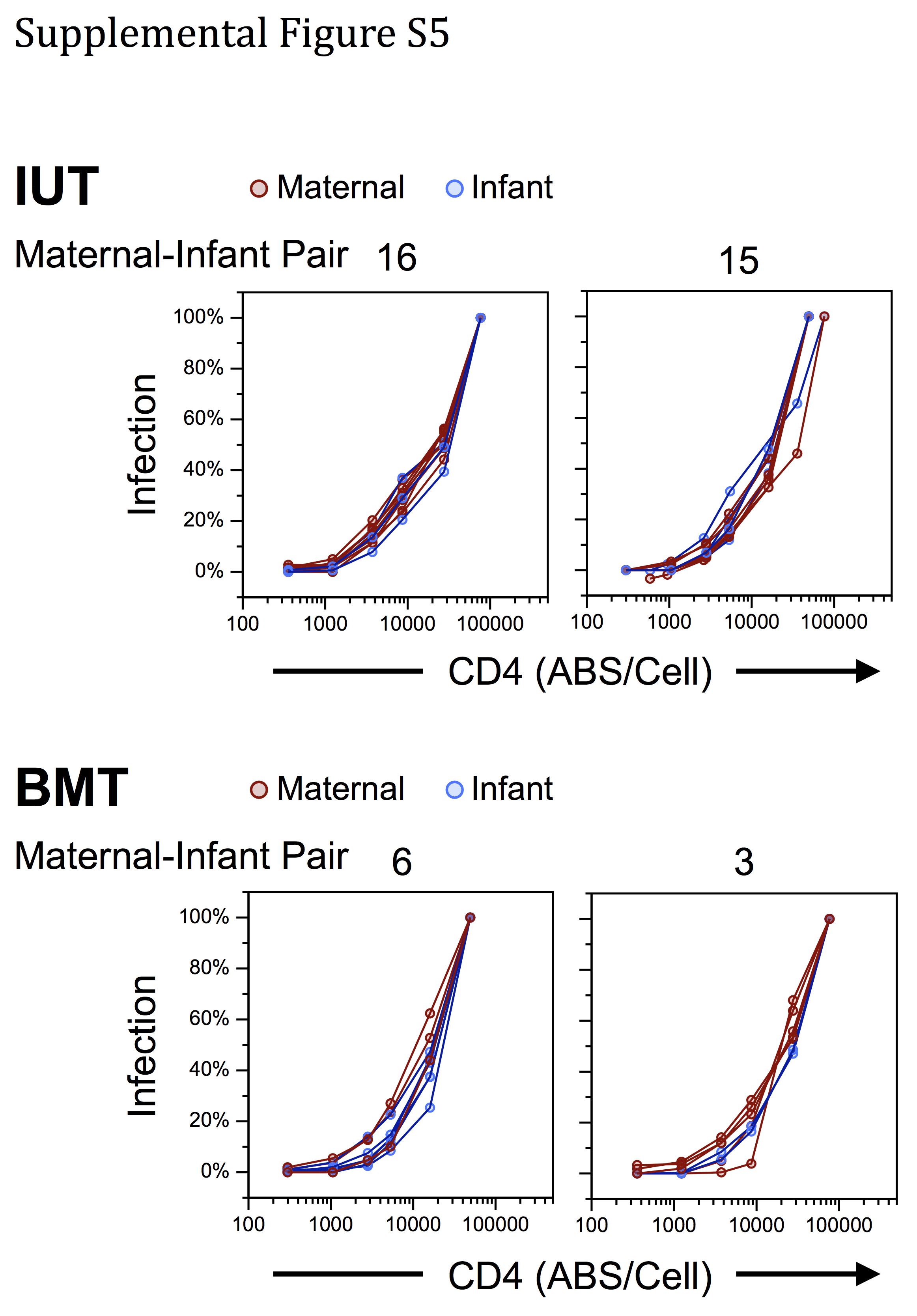

Supplement: Supplementary file 5 — Additional file 5: Figure S5.Representative CD4 infectivity curves using Affinofile cells for IUT (top) and BMT (bottom) maternal–infant pairs. Affinofile cells were induced to generate a 100-fold range of CD4 surface density (ABS/cell) and infected with 2000 IU pseudotyped virus. Percent infection was measured as the percent luciferase relative to infected and maximally induced Affinofile cells. Data shown are representative curves among 3–4 experimental replicates. [file 12977_2017_331_MOESM5_ESM.jpg]

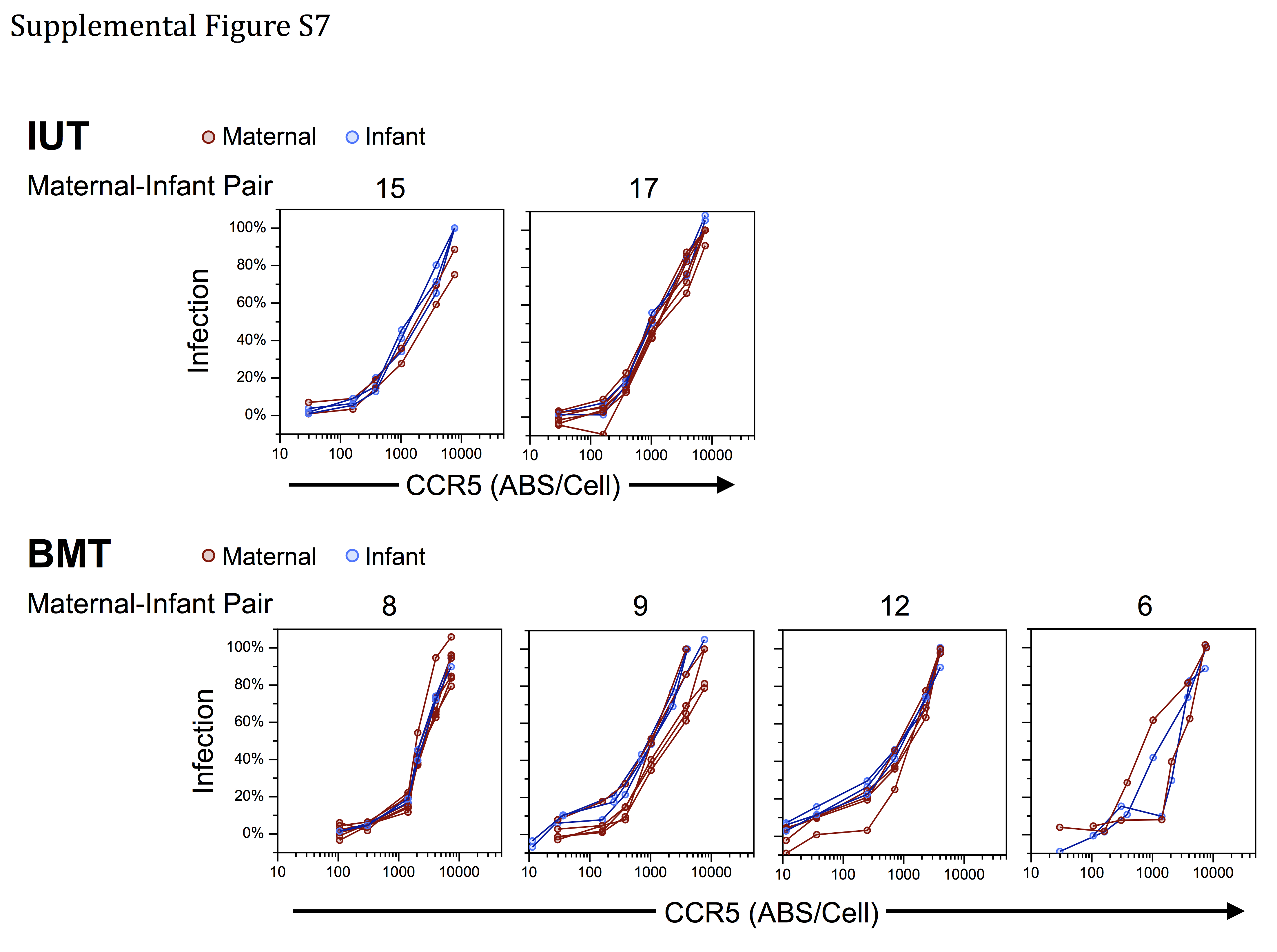

Supplement: Supplementary file 7 — Additional file 7: Figure S7.CCR5 infectivity curves using Affinofile cells for IUT (top) and BMT (bottom) maternal–infant pairs. Affinofile cells were induced to generate a 100-fold range of CCR5 surface density (ABS/cell) and infected with 2000 IU pseudotyped virus. Percent infection was measured as the percent luciferase relative to infected and maximally induced Affinofile cells. Data shown are the average of three replicates. [file 12977_2017_331_MOESM7_ESM.jpg]

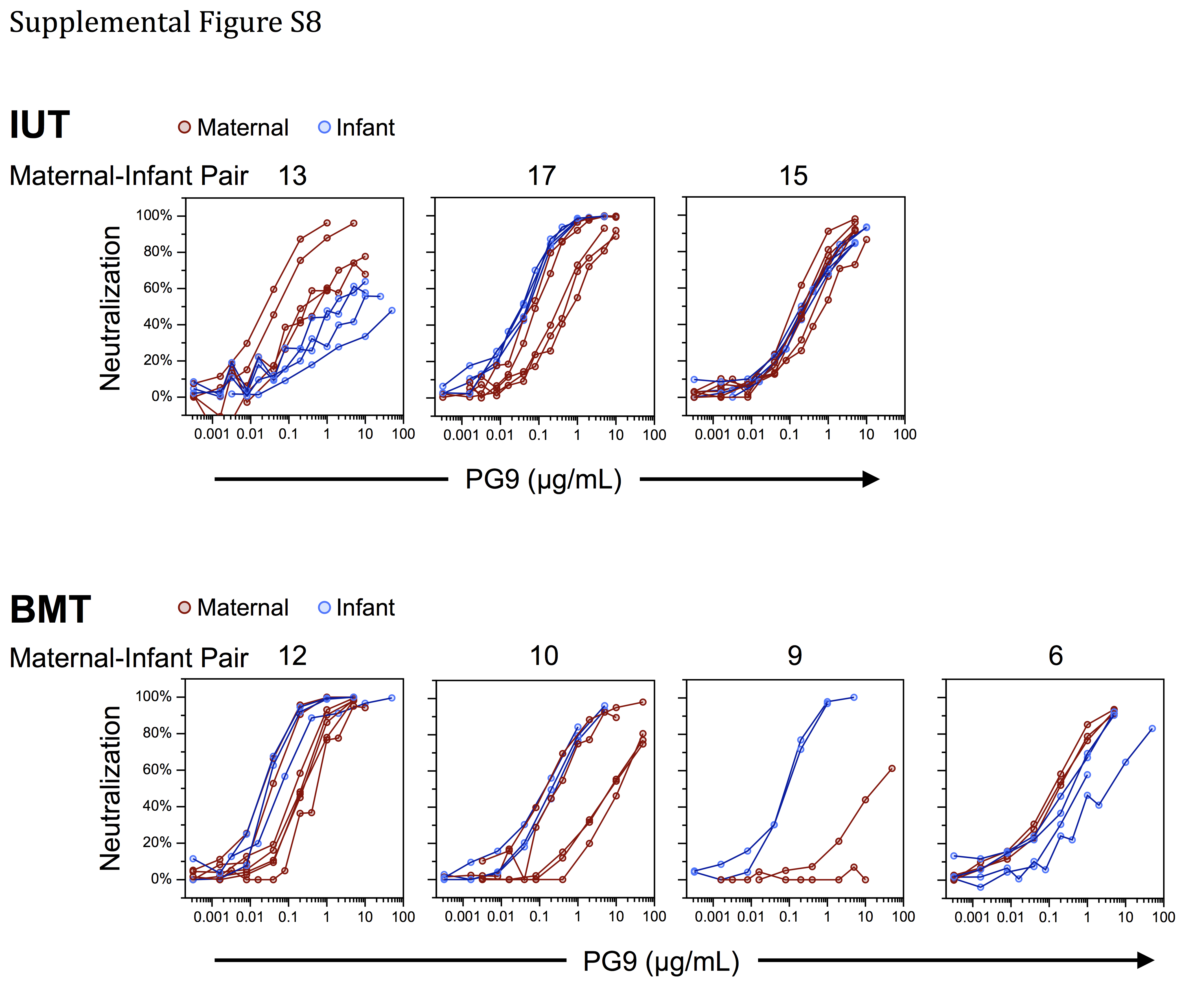

Supplement: Supplementary file 8 — Additional file 8: Figure S8.Representative PG9 neutralization curves for IUT (top) and BMT (bottom) maternal–infant pairs. Data shown are representative curves among 3–4 experimental replicates. [file 12977_2017_331_MOESM8_ESM.jpg]

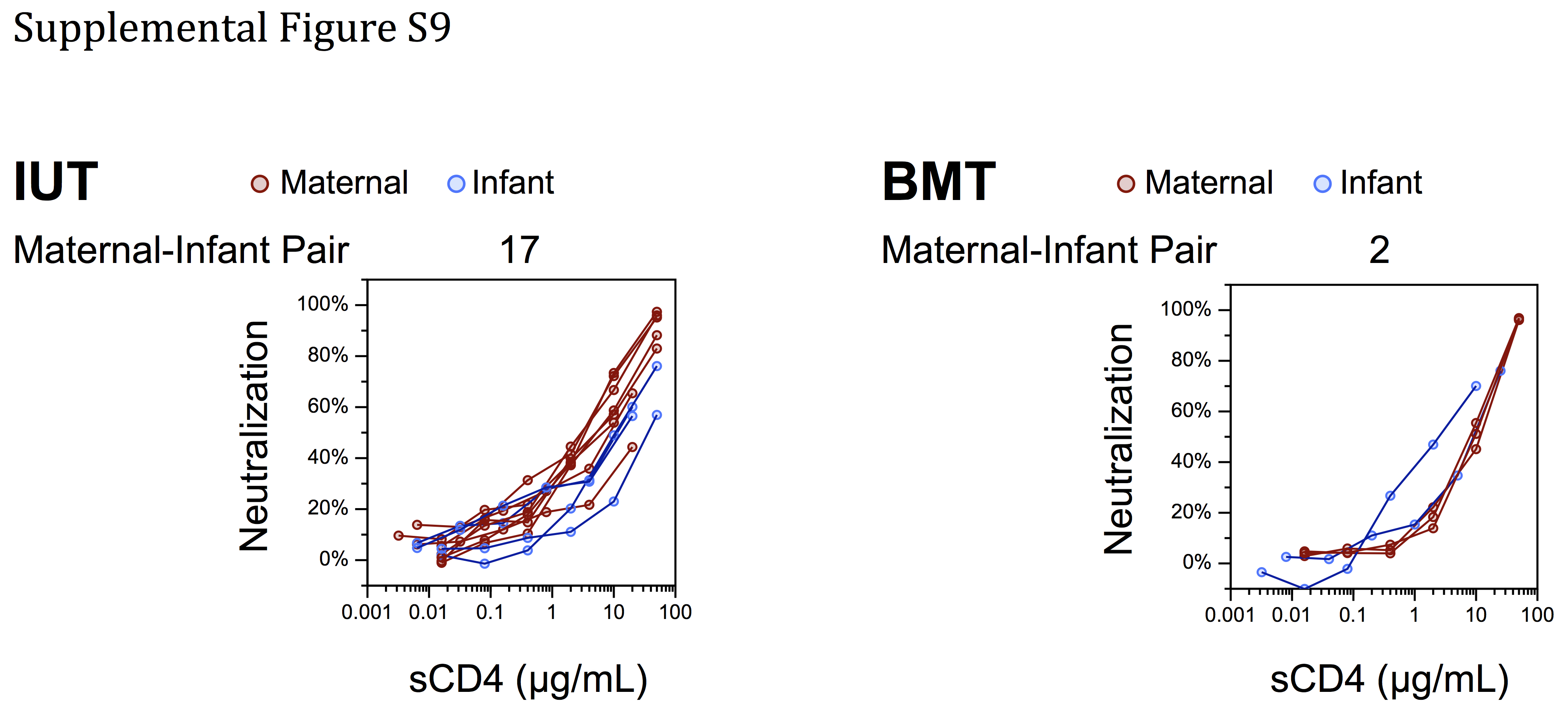

Supplement: Supplementary file 9 — Additional file 9: Figure S9.Representative sCD4 neutralization curves for IUT (left) and BMT (right) maternal–infant pairs. Data shown are representative curves among 3–4 experimental replicates. [file 12977_2017_331_MOESM9_ESM.jpg]
