# Supplementary material for: Breast milk and in utero transmission of HIV-1 select for envelope variants with unique molecular signatures
Source: Retrovirology. 2017 Jan 26;14:6. doi: 10.1186/s12977-017-0331-z (PMC5267468; doi:10.1186/s12977-017-0331-z)
Supplement: Supplementary file 2 — Additional file 2: Figure S2.Pairwise comparisons of genetic diversity within infant founder virus populations. All pairwise comparisons are shown scaled to the same genetic divergence in each panel on the X-axis, with bin intervals of 0.002, but with differentially scaled Y axes that reflect the variable number of sequences obtained from each infant. [file 12977_2017_331_MOESM2_ESM.pptx]

## Slide 1
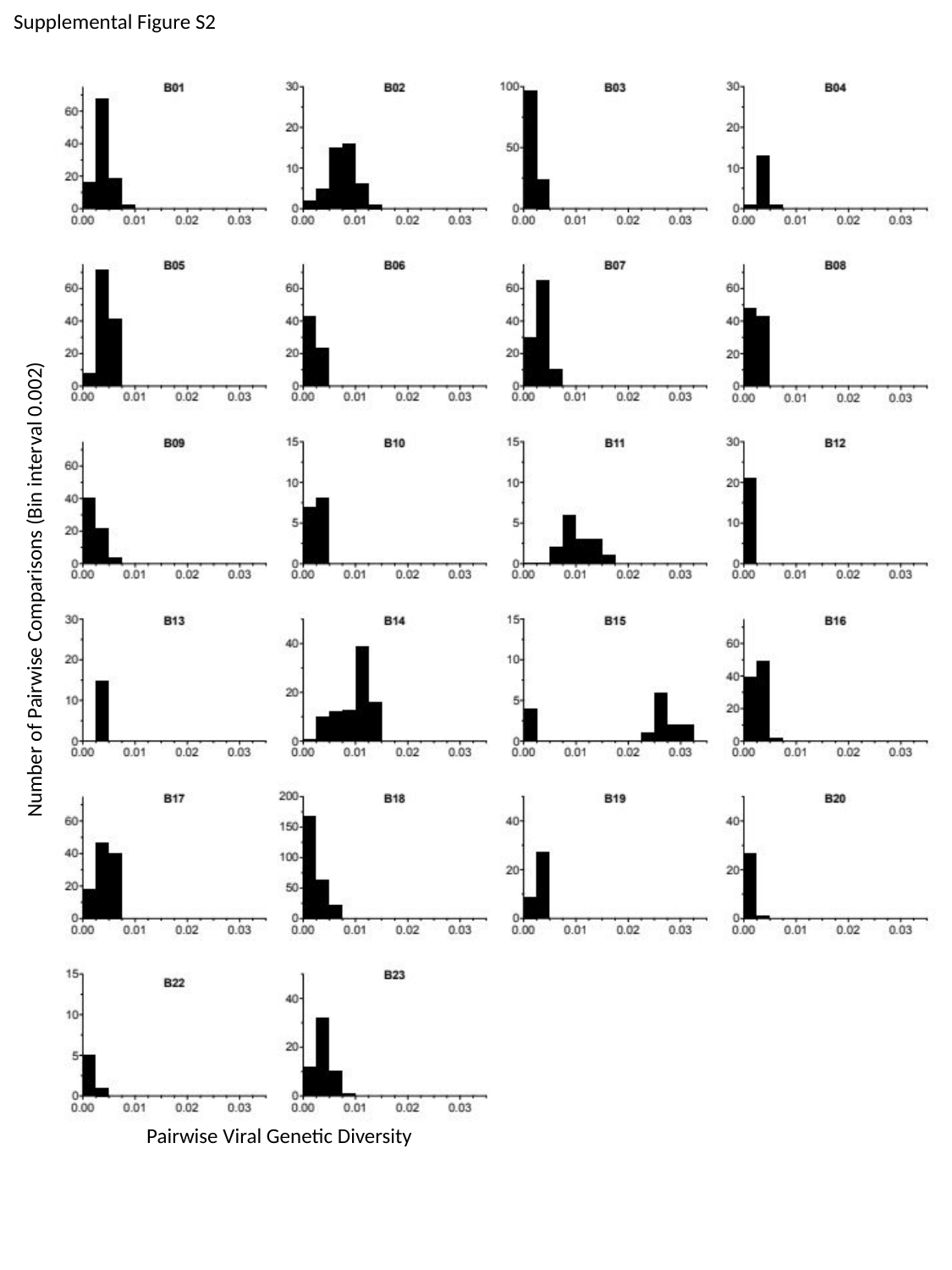

Supplemental Figure S2
Number of Pairwise Comparisons (Bin interval 0.002)
Pairwise Viral Genetic Diversity
