# Supplementary material for: Breast milk and in utero transmission of HIV-1 select for envelope variants with unique molecular signatures
Source: Retrovirology. 2017 Jan 26;14:6. doi: 10.1186/s12977-017-0331-z (PMC5267468; doi:10.1186/s12977-017-0331-z)
Supplement: Supplementary file 3 — Additional file 3: Figure S3.Phylogenetically informative mutation sites and assessment of single versus multiple virus founders in infants. (a) Number of InSites in the infant virus founder populations and the assessment of single versus multiple founders. Note that tally of these sites were only one of several criteria used to assess funder numbers (see Additional file 10: Table S1 for full list of criteria). (b, c) Phylogenetic trees and alignment of phylogenetically informative sites for sequences from the two infants in panel (a) with 6 such sites. These were the only two infants for which the data across all criteria were not unambiguous. While both had evidence of multiple variants, the presence of two founders defined by GGC versus AAA (the latter in the lower infant clade) was clearer in B19, whereas the multiple variants in B16 could have resulted from immune escape when sampled at 88 days of virus positivity. [file 12977_2017_331_MOESM3_ESM.pptx]

## Slide 1
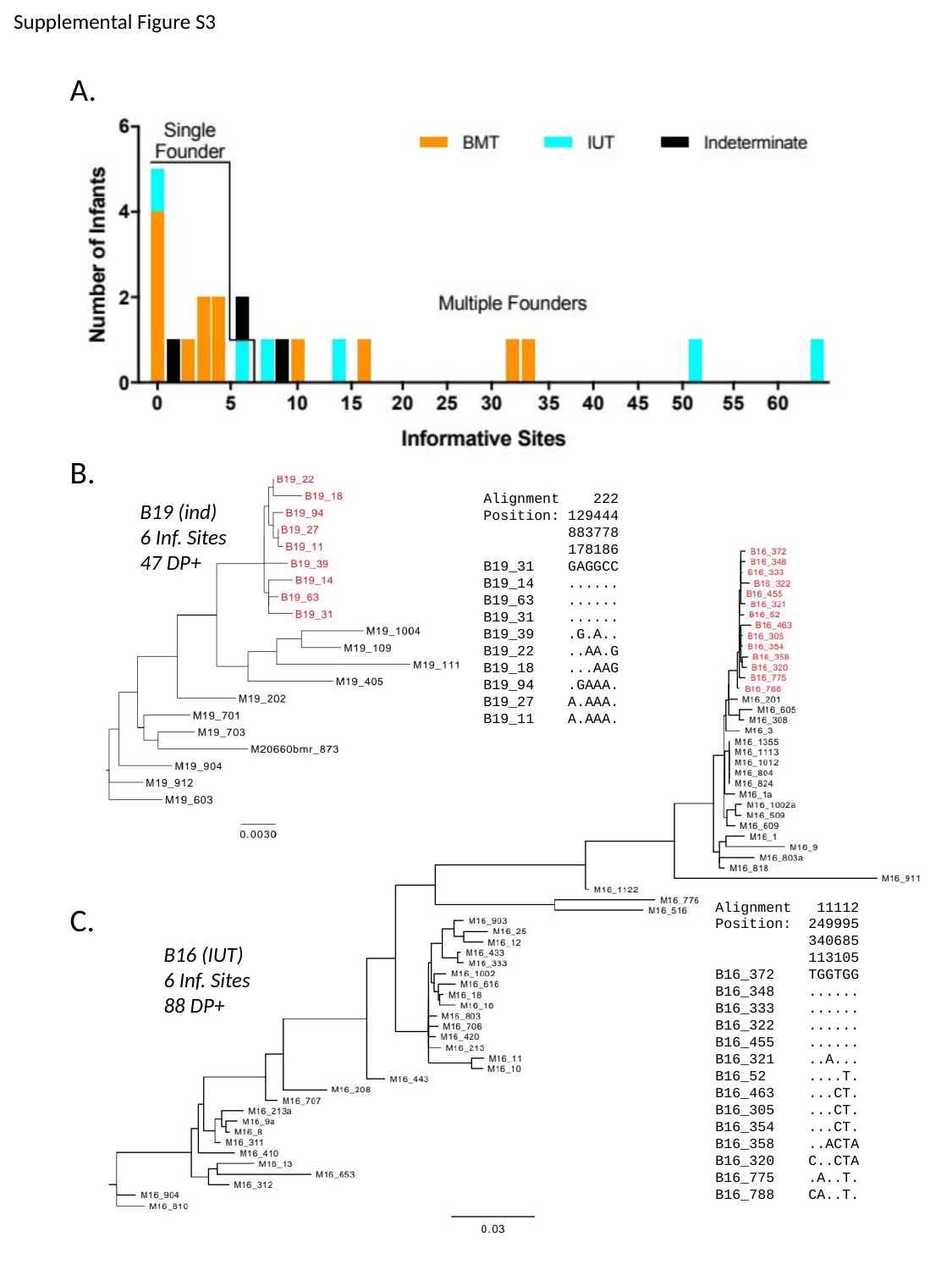

Supplemental Figure S3
A.
B.
Alignment 222
Position: 129444
 883778
 178186
B19_31 GAGGCC
B19_14 ......
B19_63 ......
B19_31 ......
B19_39 .G.A..
B19_22 ..AA.G
B19_18 ...AAG
B19_94 .GAAA.
B19_27 A.AAA.
B19_11 A.AAA.
B19 (ind)
6 Inf. Sites
47 DP+
Alignment 11112
Position: 249995
 340685
 113105
B16_372 TGGTGG
B16_348 ......
B16_333 ......
B16_322 ......
B16_455 ......
B16_321 ..A...
B16_52 ....T.
B16_463 ...CT.
B16_305 ...CT.
B16_354 ...CT.
B16_358 ..ACTA
B16_320 C..CTA
B16_775 .A..T.
B16_788 CA..T.
C.
B16 (IUT)
6 Inf. Sites
88 DP+
