# Supplementary material for: Breast milk and in utero transmission of HIV-1 select for envelope variants with unique molecular signatures
Source: Retrovirology. 2017 Jan 26;14:6. doi: 10.1186/s12977-017-0331-z (PMC5267468; doi:10.1186/s12977-017-0331-z)
Supplement: Supplementary file 10 — Additional file 10: Table S1.Identification of founder variants establishing infection. Infant infections that were inferred to be associated with single (S) or multiple (M) founder variants are shown, along with data used to make these determinations. Given the varying time between the first PCR-positive HIV test and virus population sampling in the infected infant, the wide range in the number of maternal and infant viral sequences sampled, and the unknown impact of early immune responses in selecting for mutations in the virus population, multiple criteria were considered when assessing whether transmission was associated with single or multiple founder variants. These criteria included (i) the number of phylogenetically informative mutation sites (InSites) computed by DIVEIN (doi: 10.2144/000113370), (ii) the minimum number of viral sublineages (multiple sequences sharing the same InSites), (iii) the estimated number of shared mutations defining these sublineages, (iv) whether insertions or deletions (InDels) were detected and shared across different sequences, (v) whether probable recombination was observed between sequences, and (vi) the number of maternal sequences found to represent the most recent common ancestor (MRCA) of the infant’s infection in phylogenetic trees. Multiple founders were inferred when the infant virus population emerged from more than a single MRCA in the mother, and/or when sublineages were detected that differed by 4 or more mutations (including InDels). Supporting these inferences were the detection of recombinants between sublineage sequences. The number of sublineages and mutations defining these sublineages were in some cases not precisely defined due to recombination. While in utero transmission showed a higher incidence of multiple founder virus outgrowth (67% in the IUT group versus 31% in the BMT group), our sampling was insufficient to determine a statistically-significant difference (p = 0.319, Fisher’s exact test). [file 12977_2017_331_MOESM10_ESM.doc]

Table S1

*Only a single maternal sequence was available for MRCA determination.

**Table S1:** Identification of founder variants establishing infection. Infant infections that were inferred to be associated with single (S) or multiple (M) founder variants are shown, along with data used to make these determinations. Given the varying time between the first PCR-positive HIV test and virus population sampling in the infected infant, the wide range in the number of maternal and infant viral sequences sampled, and the unknown impact of early immune responses in selecting for mutations in the virus population, multiple criteria were considered when assessing whether transmission was associated with single or multiple founder variants. These criteria included (i) the number of phylogenetically informative mutation sites (InSites) computed by DIVEIN (DOI: 10.2144/000113370), (ii) the minimum number of viral sublineages (multiple sequences sharing the same InSites), (iii) the estimated number of shared mutations defining these sublineages, (iv) whether insertions or deletions (InDels) were detected and shared across different sequences, (v) whether probable recombination was observed between sequences, and (vi) the number of maternal sequences found to represent the most recent common ancestor (MRCA) of the infant’s infection in phylogenetic trees. Multiple founders were inferred when the infant virus population emerged from more than a single MRCA in the mother, and/or when sublineages were detected that differed by 4 or more mutations (including InDels). Supporting these inferences were the detection of recombinants between sublineage sequences. The number of sublineages and mutations defining these sublineages were in some cases not precisely defined due to recombination. While *in utero* transmission showed a higher incidence of multiple founder virus outgrowth (67% in the IUT group versus 31% in the BMT group), our sampling was insufficient to determine a statistically-significant difference (p = 0.319, Fisher’s exact test).
